# Maternal separation leads to dynamic changes of visceral hypersensitivity and fecal metabolomics from childhood to adulthood

**DOI:** 10.1038/s41598-023-34792-7

**Published:** 2023-05-11

**Authors:** Xiaolong Chen, Chenmin Hu, Chenxi Yan, Enfu Tao, Zhenya Zhu, Xiaoli Shu, Rui Guo, Mizu Jiang

**Affiliations:** 1 Pediatric Endoscopy Center and Gastrointestinal Laboratory, Children’s Hospital, Zhejiang University School of Medicine, National Clinical Research Center for Child Health, National Children’s Regional Medical Center, Hangzhou, 310052 China; 2 Department of Pediatrics, The First People’s Hospital of Jiashan, Jiashan, 314100 China; 3 Department of Gastroenterology, Children’s Hospital, Zhejiang University School of Medicine, National Clinical Research Center for Child Health, National Children’s Regional Medical Center, Hangzhou, 310052 China

**Keywords:** Chronic pain, Enteric neuropathies, Irritable bowel syndrome

## Abstract

We assessed dynamic changes in visceral hypersensitivity and fecal metabolomics through a mouse model of irritable bowel syndrome (IBS) from childhood to adulthood. A mouse model of IBS was constructed with maternal separation (MS) in early life. Male mice aged 25, 40, and 70 days were used. Visceral sensitivity was assessed by recording the reaction between the abdominal withdrawal reflex and colorectal distension. Metabolomics was identified and quantified by liquid chromatography-tandem mass spectrometry. The visceral sensitivity of the MS group was significantly higher than that of the non-separation (NS) group in the three age groups. The top four fecal differential metabolites in the different age groups were lipids, lipid molecules, organic heterocyclic compounds, organic acids and derivatives, and benzenoids. Five identical differential metabolites were detected in the feces and ileal contents of the MS and NS groups at different ages, namely, benzamide, taurine, acetyl-L-carnitine, indole, and ethylbenzene. Taurine and hypotaurine metabolism were the most relevant pathways at P25, whereas histidine metabolism was the most relevant pathway at P40 and P70. Visceral hypersensitivity in the MS group lasted from childhood to adulthood. The different metabolites and metabolic pathways detected in MS groups of different ages provide a theoretical basis for IBS pathogenesis.

## Introduction

Irritable bowel syndrome (IBS) is characterized by functional bowel disease with abdominal discomfort or pain with changes in bowel habits. Population based epidemiological studies provide an approximation of the true prevalence rate, which ranges from 5% to 10% in most geographical regions^1^. The pathogenesis and pathophysiology of IBS are complex and not completely clear at present. Potential pathogenic factors include heredity, visceral hypersensitivity, gastrointestinal motility, brain gut axis disorder, neuropeptide and hormone levels, inflammatory changes, changes in intestinal microbiota, and so on^2–4^.

Early adverse life events (EALs) adversely affect the health of the body and increase the risk of functional gastrointestinal diseases such as IBS in later life^5^. Neonatal maternal separation (MS) in rodents is a well-known animal model of early life stress which has been shown to cause a variety of gastrointestinal dysfunctions, including hyperalgesia to colorectal distension^6,7^, increased permeability of the colonic mucosa^8^, and changes in colonic motility^9^. However, few studies have been conducted on the dynamic change process of IBS from early life to adulthood.

An increasing number of studies have shown that intestinal flora and metabolites are an important part of the intestinal mucosa micro-ecosystem, and intestinal mucosa micro-ecosystem changes are closely related to the development of IBS^10,11^. Gut microbiota and its metabolites play a role in a variety of host metabolic pathways, including cellular signaling, energy transfer, and immune inflammation^12^. At present, there are many studies on the intestinal microbiome of IBS, but few on metabolomics, especially the dynamic changes in IBS metabolomics.

Metabolome is the final product of complex interactions among genome, transcriptome, proteome and environment. For qualitative and quantitative analyses of dynamic responses, metabolic metabolites can be integrated into living systems and environmental change^13^. Studies have shown that stool specimens can be used to evaluate gut microbes for metabolic effects^14^. However, Lkhagva et al.^15^ concluded that the microbiome in different locations of the gastrointestinal tract is completely different, and the diversity of microbiome composition in the ileum may be the highest. It is worth noting whether the metabolites in different locations of the gastrointestinal tract are also different.

Non-targeted metabolomics can be applied to fully clinical cohort in combination with mechanism research through animal models, which can be applied to human disease-related characterization of gut microbes and their metabolites^16^. Liquid chromatography-tandem mass spectrometry (LC–MS/MS) with high sensitivity, high selectivity, high flux, and detectable residual compounds is commonly used for rapid and highly sensitive quantitative analysis^13^.

In this study, a mouse model of MS^17^ was used to investigate the pathology of IBS ileum content and fecal metabolomic changes by studying the dynamic effects of visceral hypersensitivity in childhood (P25), adolescent (P40), and adult (P70)^18^ mice after maternal separation in early life, as well as the changes in differential metabolites and metabolic pathways of the ileal contents and feces. We aim to explore possible biomarkers and metabolic pathways of IBS.

## Materials and methods

### Animal preparation and experimental design

The mice used in our experiment were C57BL/6J, which were purchased from the Experimental Animal Center of Zhejiang University (Hangzhou, China). We prepared male and female mice (8 weeks old) with a ratio of 1:2, and placed them in cages (cage size 318 × 202 × 135 mm) on a 12-h light and shade cycle (lights on at 9 am, lights off at 9 pm), free access to food and water, ambient temperatures set at 25–26 °C, humidity 50 ± 5%, ammonia concentration < 20 PPM, and ventilation rate 8–15 times/h. Pregnant mice were maintained separately after mating. Pregnancy mouse experiments were conducted on deliveries for the same day. MS group pups born between 2 and 14 days were separated from the dams for three hours daily (9 to 12 am) and placed in a plastic box fitted with wood-chip bedding (box size 180 × 130 × 55 mm), temperature controlled at 26 ± 2 °C, and bedding was changed once a week after separated mice were returned to dams. On the other hand, non-separation (NS) group pups were not given any interventional treatment, and all pups were weaned on postnatal day 22 (P22). On P22, the female pups were removed to avoid periodic estrogen interference. Excluding female mice and mice that died at birth, 36 mice could be used in the experiment. The male newborn mice were standardized into 6 pups per fetus and randomly divided into MS group (*n* = 18) and NS group (*n* = 18). Pups were marked for easy identification. On P25, P40, and P70, fecal and ileal content samples were taken from six randomly selected mice in each group for the metabolomic study. Feces were collected over a 24-h period prior to euthanasia. The mice were anesthetized by inhaling 1.5% isoflurane for 1 min. After euthanasia, the mice were killed using cervical dislocation, and the gut was prepared by dissection, and the scissors and tweezers used in the experiment were autoclaved and dried in advance. The ileal contents were collected, and all samples were placed in RNA free eppendorf tubes (1.5 ml) and held at − 80 °C prior to analysis.

### Visceral sensitivity assessment

On P25, P40, and P70, six mice in each group were randomly selected for evaluation of visceral sensitivity by measuring the response of the abdominal withdrawal reflex (AWR) to colorectal distension (CRD)^7,19^. We had invented a novel and exquisite inflatable balloon (1 cm in length and 0.3–0.5 cm in diameter), which had been previously published^7^. Different specifications of expansion balloon were used according to the age and weight of mice. A 3 mm diameter balloon was used for the AWR score of P25 and P40 mice, while a 5 mm diameter balloon was used for the AWR score of P70 mice. Before the experiment, the mice were acclimatized to the environment for 15 min. Under the anesthesia of 1.5% isoflurane (RWD Life Science, Shenzhen, China), the balloon was inserted into the rectum 1.0 cm from the anus and fixed at the bottom of the tail. The mice were then placed in a hollow cylinder made of transparent acrylic acid and allowed to fully recover from anesthesia and balloon adaptation for 30 min. The mice were fixed to the tee so that it cannot move back and forth, but can move up and down. The AWR score was 0–4: 0, there was no behavioral response to CRD; 1, the head movement is short, and then there is no movement; 2, abdominal muscle contraction; 3, raise the abdomen; 4, arch and lift the pelvic structure. In order to measure the pressure threshold of AWR scores 1, 2, 3, and 4, the colorectal balloon gradually expanded from 0 mmHg pressure to the maximum pressure, showing different degrees of pain behavior (0–4), and recorded the real-time pressure in a pre-made form. When recording, balloon inflation was stopped, but the pressure was maintained. In this way, pressure thresholds for AWR scores 1, 2, 3, and 4 were determined and continuously recorded. Repeated the measurement after 5 min of rest. Measured the AWR of the balloon under different pressure (10, 20, 30, 40, 50, 60, 70, and 80 mmHg). Each pressure was maintained for 5 s, and then the balloon deflated rapidly and completely. After 20 s of rest, carried out the next pressurization. Similarly, repeated the measurement after 5 min of rest. The pressure was always from lowest to highest. Accurate records were obtained by two investigators blinded to randomization^7^.

### Metabolite extraction

The collected ileal contents and feces were thawed on ice, and metabolites were extracted using 50% methanol buffer, following which 20 μL of the sample was extracted with 120 μL of precooled 50% methanol, vortexed for 1 min, and incubated at room temperature for 10 min; the extraction mixture was then stored overnight at − 20 °C. After centrifugation at 4000 × g for 20 min, the supernatants were transferred to new 96-well plates. The samples were stored at − 80 °C before the LC–MS/MS analysis. Pooled quality control (QC) samples were prepared by combining 10 μL of each extraction mixture^20^.

### LC–MS/MS analysis conditions

All samples were acquired using an LC–MS/MS system followed by machine ordering. All chromatographic separations were performed using a Thermo Scientific UltiMate 3000 HPLC system. An ACQUITY UPLC BEH C18 column (100 mm × 2.1 mm, 1.8 µm, Waters, UK) was used for reversed-phase separation. The column oven was maintained at 35 °C. The flow rate was 0.4 ml/min and the mobile phase consisted of solvent A (0.1% formic acid in water) and solvent B (0.1% formic acid in acetonitrile). Gradient elution conditions were set as follows: 0–0.5 min, 5% solvent B; 0.5–7 min, 5% to 100% solvent B; 7–8 min, 100% solvent B; 8–8.1 min, 100% to 5% solvent B; 8.1–10 min, 5% solvent B. The injection volume for each sample was 4 µL.

A high-resolution tandem mass spectrometer (Q-Exactive, Thermo Scientific) was used to detect metabolites eluted from the column. Q-Exactive was operated for both the positive and negative ion modes. Precursor spectra (70–1050 m/z) were collected at 70,000 resolution to hit an AGC target of 3e6. The maximum injection time was set as 100 ms. The top three configurations for acquiring data were set at the DDA mode. Fragment spectra were collected at 17,500 resolution to hit an AGC target of 1e5, with a maximum injection time of 80 ms. To evaluate the stability of LC–MS/MS during the entire acquisition, a QC sample (a pool of all samples) was acquired after every 10 samples.

### Metabolomics data analysis

The acquired LC–MS/MS data pretreatments, including peak picking, peak grouping, retention time correction, second peak grouping, and annotation of isotopes and adducts, were performed using the XCMS software. LC–MS/MS raw data files were converted into mzXML format and then processed using the XCMS, CAMERA, and metaX^21^ toolbox implemented with R software. Each ion was identified by combining retention time (RT) and m/z data. The intensities of each peak were recorded, and a three-dimensional matrix containing arbitrarily assigned peak indices (retention time-m/z pairs), sample names (observations), and ion intensity information (variables) was generated. The online kyoto encyclopedia of genes and genomes (KEGG) and human metabolome database (HMDB) databases were used to annotate the metabolites by matching the exact molecular mass data (m/z) of the samples with those from the database. If the mass difference between the observed and the database value was less than 10 ppm, the metabolite was annotated, and the molecular formula of the metabolites was further identified and validated by isotopic distribution measurements. We also used an in-house fragment spectrum library of metabolites to validate metabolite identification.

The peak intensity data were preprocessed using the metaX software. The features detected in less than 50% QC samples and 80% biological samples were eliminated, and the remaining missing value peaks were estimated with a k-nearest neighbor algorithm to further improve data quality. Principal component analysis (PCA) was used to detect outliers and evaluate batch effects. Quality control-based loss signal correction was fitted to the injection sequence of the QC data to minimize the drift of the signal strength over time. In addition, the relative standard deviation of the metabolic characteristics of all QC samples was calculated, and those > 30% were excluded. Using the metaX R package for data preprocessing, statistical analysis (univariate and multivariate analysis), significant differences in metabolite screening based on the multivariate analysis partial further squares method-discriminant analysis (PLS-DA) model for the first two principal components of variable important for the projection (VIP) multiples differences in values, and univariate analysis fold change, (FC) and *t*-test (Student's *t*-test) *P*-value results to screen for different metabolites. Univariate analysis of differential metabolites was required to meet the following requirements: FC ≥ 2 or FC ≤ 1/2, *P* ≤ 0.05. Conditions to be met simultaneously in multivariate analysis include: VIP ≥ 1, FC ≥ 2, or FC ≤ 1/2, *P* ≤ 0.05. The KEGG database was used to link metabolites with metabolic pathways, and the most relevant pathways were identified by MetaboAnalyst 5.0 comprehensive enrichment analysis and pathway analysis. The larger the value of path influence, the more meaningful it was. Combined with the *P* value, the path in the upper right corner of the bubble chart was the most reliable^22^.

### Statistical analysis

The sample size was analyzed using Power Analysis and Sample Size (PASS 15.0). The distribution of data was analyzed by Kolmogorov–Smirnov test. Data were shown to fit a normal distribution and were expressed as mean ± SD. Differences between two groups were determined by two-way repeated measures ANOVA with Sidak’s multiple comparisons test. All data were analyzed by IBM Statistical Package for the Social Sciences (SPSS), version 25 (IBM SPSS Statistics). Statistical significance was set at *P* < 0.05.

### Ethical approval

All experimental protocols were approved by the Zhejiang University Ethics Committee for Animal Research (approval number ZJU20230025). All methods were carried out in accordance with relevant guidelines and regulations. All methods are reported in accordance with ARRIVE guidelines.

## Results

### Comparison of visceral sensitivity between MS group and NS group at different ages

**Figure 1:**
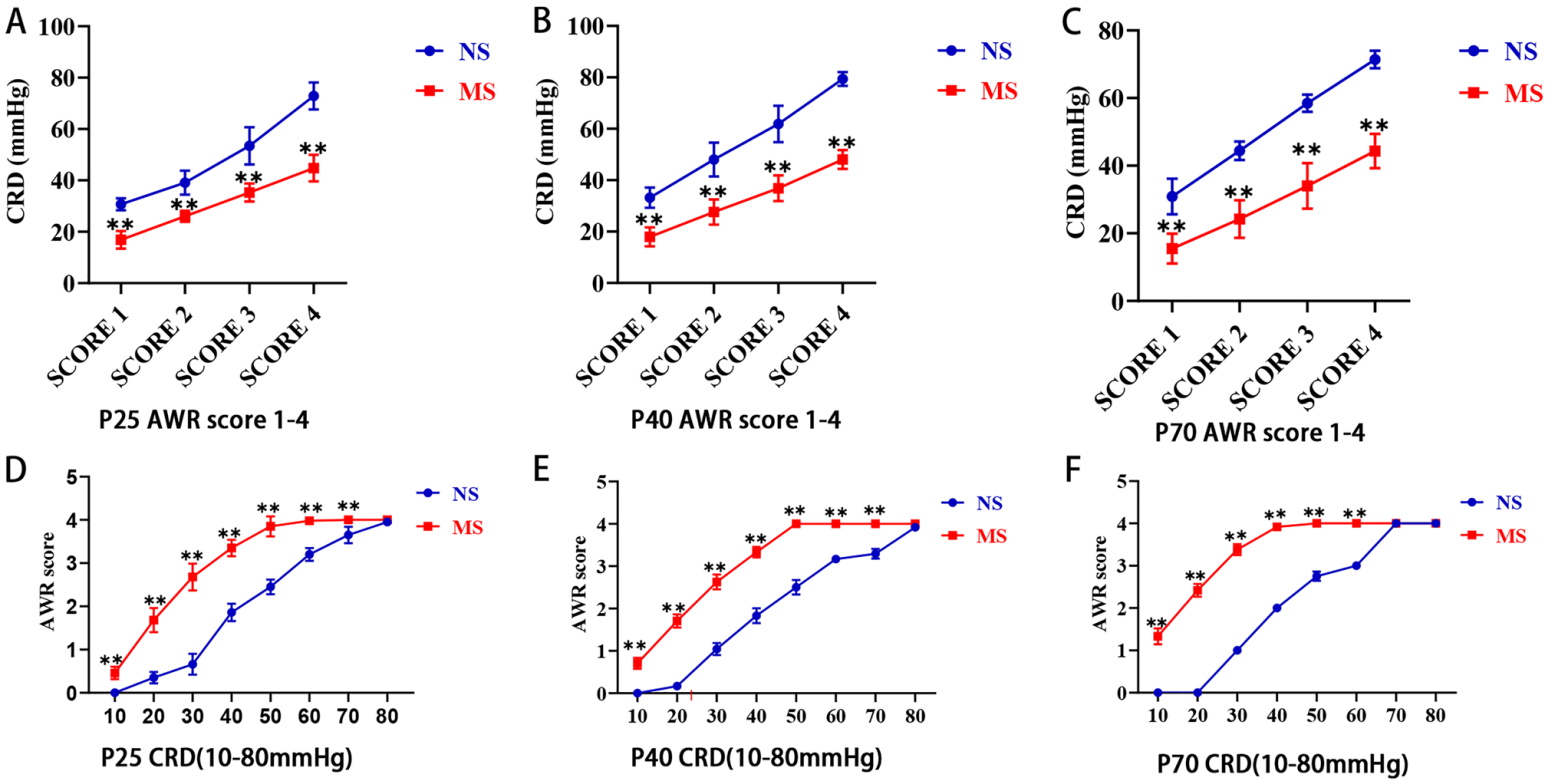
Comparison of visceral sensitivity between MS group and NS group at different ages. (**A**)–(**C**) comparison of CRD thresholds of different AWR scores. (**D**)–(**F**) comparison of AWR scores under different CRD pressures. MS, maternal separation; NS, non-separation; CRD, colorectal dilatation; AWR, abdominal withdrawal reflex. Differences between two groups were determined by two-way repeated measures ANOVA with Sidak’s multiple comparisons test. ** *P* < 0.01. MS, *n* = 6; NS, *n* = 6.

An IBS model of visceral hyperalgesia was successfully established for the MS group in this study, and we found that visceral hypersensitivity in the MS group persisted from childhood to adulthood. On P25, the threshold values of CRD pressure in the MS groups were significantly lower than that of NS groups at an abdominal withdrawal test score of 1,2,3,4. *P* all < 0.01 (Fig. 1A); Similar to the results of P25, the CRD threshold of AWR scores in the MS groups at P40 and P70 were significantly lower than that of the NS groups, *P* all < 0.01 (Fig. 1B,C). Then the AWR scores of two groups at different CRD pressures (10–80 mmHg) during three periods were measured. The data were analyzed using two-way ANOVA (EALs, Pressure) followed by Sidak’s multiple comparisons test. On P25, the AWR score in the MS groups were significantly higher than that of NS groups at 10, 20, 30, 40, 50, 60, 70 mmHg pressure of CRD *(P* < 0.01), but 80 mmHg pressure of CRD (*P* > 0.05) (Fig. 1D), and the interaction of two variations both EALs and CRD pressure had reached significance, F(7,160) = 80.57, *P* < 0.0001; the main effect of EALs had reached significance , F(1,160) = 1361, *P* < 0.0001; the main effect of CRD pressure had reached significance, F(7,160) = 1364, *P* < 0.0001. On P40, the AWR score in the MS groups were significantly higher than that of NS groups at 10, 20, 30, 40, 50, 60, 70 mmHg pressure of CRD (*P* < 0.01), but 80 mmHg pressure of CRD (*P* > 0.05) (Fig. 1E), and the interaction of two variations both EALs and CRD pressure had reached significance, F(7,176) = 140.9, *P* < 0.0001; the main effect of EALs had reached significance, F(1,176) = 4111, *P* < 0.0001; the main effect of CRD pressure had reached significance, F(7,176) = 3329, *P* < 0.0001. On P70, the AWR score in the MS groups were significantly higher than that of NS groups at 10, 20, 30, 40, 50, 60 mmHg pressure of CRD *(P* < 0.01), but 70, 80 mmHg pressure of CRD (*P* > 0.05) (Fig. 1F), and the interaction of two variations both EALs and CRD pressure had reached significance, F(7,168) = 872.4, *P* < 0.0001; the main effect of EALs had reached significance, F(1,168) = 12,901, *P* < 0.0001; the main effect of CRD pressure had reached significance, F(7,168) = 6200, *P* < 0.0001.

### Comparison of fecal differential metabolites between MS group and NS group at different ages

**Figure 2:**
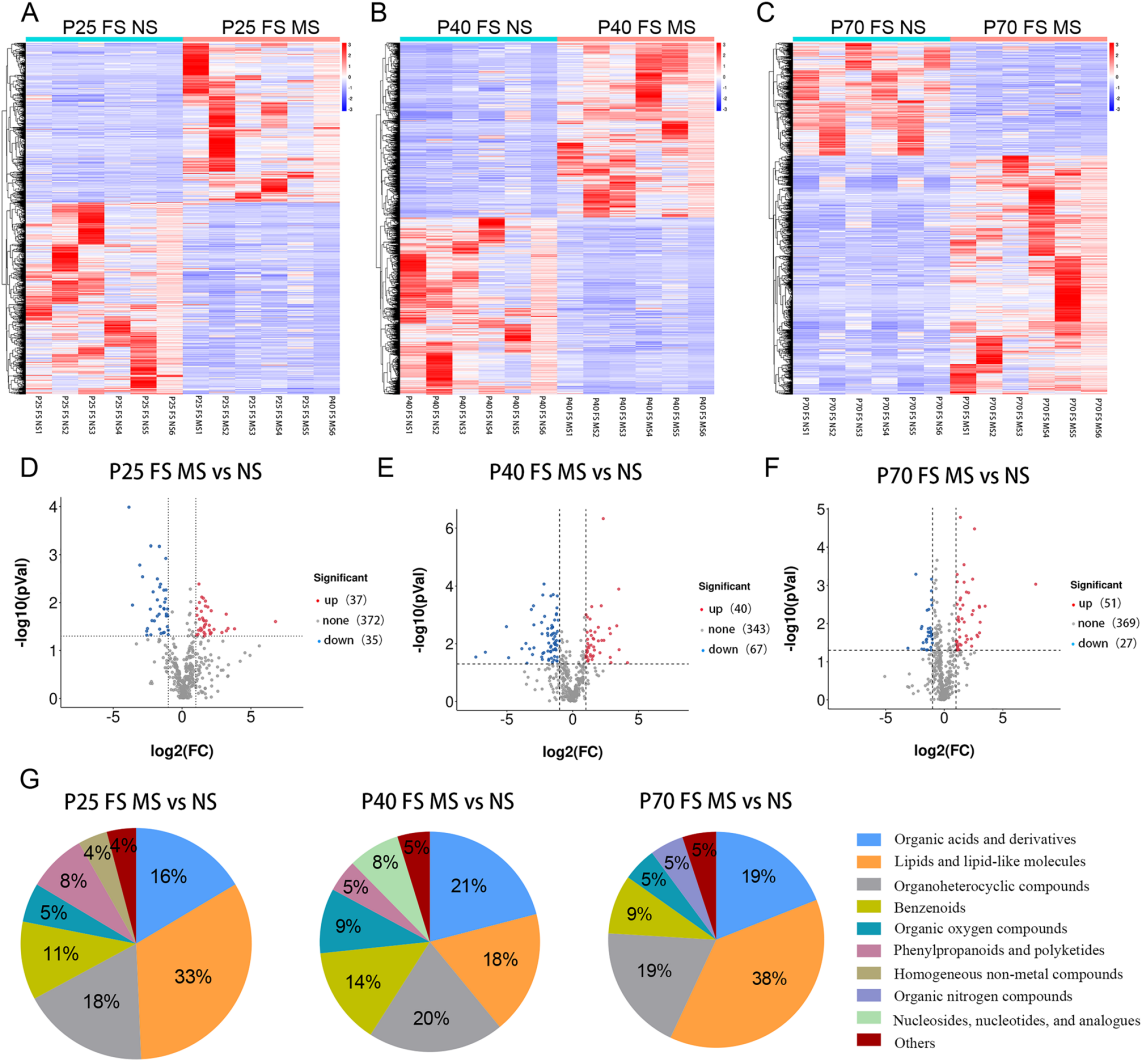
Comparison of fecal differential metabolites between MS group and NS group at different ages. (**A**)–(**C**) showed the ionic strengths detected in the fecal samples of the two groups on P25, P40, and P70. Each row represented an ion, and each column represented a sample. Different colors represented different intensities, where red indicated high intensity and blue indicated low intensity. (**D**)–(**F**) respectively represented the metabolites detected in the fecal samples of the two groups on P25, P40, and P70. The red dots represented significantly upregulated differential metabolites, the blue dots represented significantly downregulated differential metabolites, and gray dots represented metabolites without differences between the two groups. (**G**) represented the classification composition of the differential metabolites detected in the stool samples of the two groups at P25, P40, and P70. MS, maternal separation. NS, non-separation. FS, feces.

The ion cluster heatmap of fecal metabolites in the MS and NS groups at three ages were distinguishable, indicating that the ion intensity of metabolites in the two groups at different ages changed significantly (Fig. 2A–C). At P25, a total of 444 metabolites were detected in the fecal samples of the MS group compared with the NS group, and 72 metabolites were different (37 were upregulated and 35 were downregulated) (Fig. 2D). A total of 450 and 447 metabolites were detected on P40 and P70, of which 107 (40 were upregulated and 67 were downregulated) and 78 (51 were upregulated and 27 were downregulated) were different metabolites (Fig. 2E,F). At P25, the top four differential metabolites were lipids and lipid molecules (33%), organic heterocyclic compounds (18%), organic acids and derivatives (16%), and benzene ring compounds (11%) (Fig. 2G). At P40 and P70, the classification of the top four was the same as that of P25 (Fig. 2G). Among the three age groups, the most and least differential metabolites were identified at P40 (puberty) and P25 (infancy), respectively. Nine metabolites differed between the three age groups, namely, 1-palmitoylglycerol phosphocholine, 2-keto-4-methylthiobutyric acid, benzamide, styrene, taurine, porphobilinogen, ethylbenzene, acetyl-L-carnitine, and indole. Among these, 1-palmitoylglycerol phosphocholine was upregulated in the three age groups, and 2-keto-4-methylthiobutyric acid was downregulated in all three age groups. Styrene, taurine, ethylbenzene, and indole were downregulated at P25 and upregulated at P40 and P70. Acetyl-L-carnitine was upregulated at P25 and downregulated at P40 and P70. Benzamide and porphobilinogen were downregulated at P25 and upregulated at P40 and P70 (Fig. 2D,E,F).

**Figure 3:**
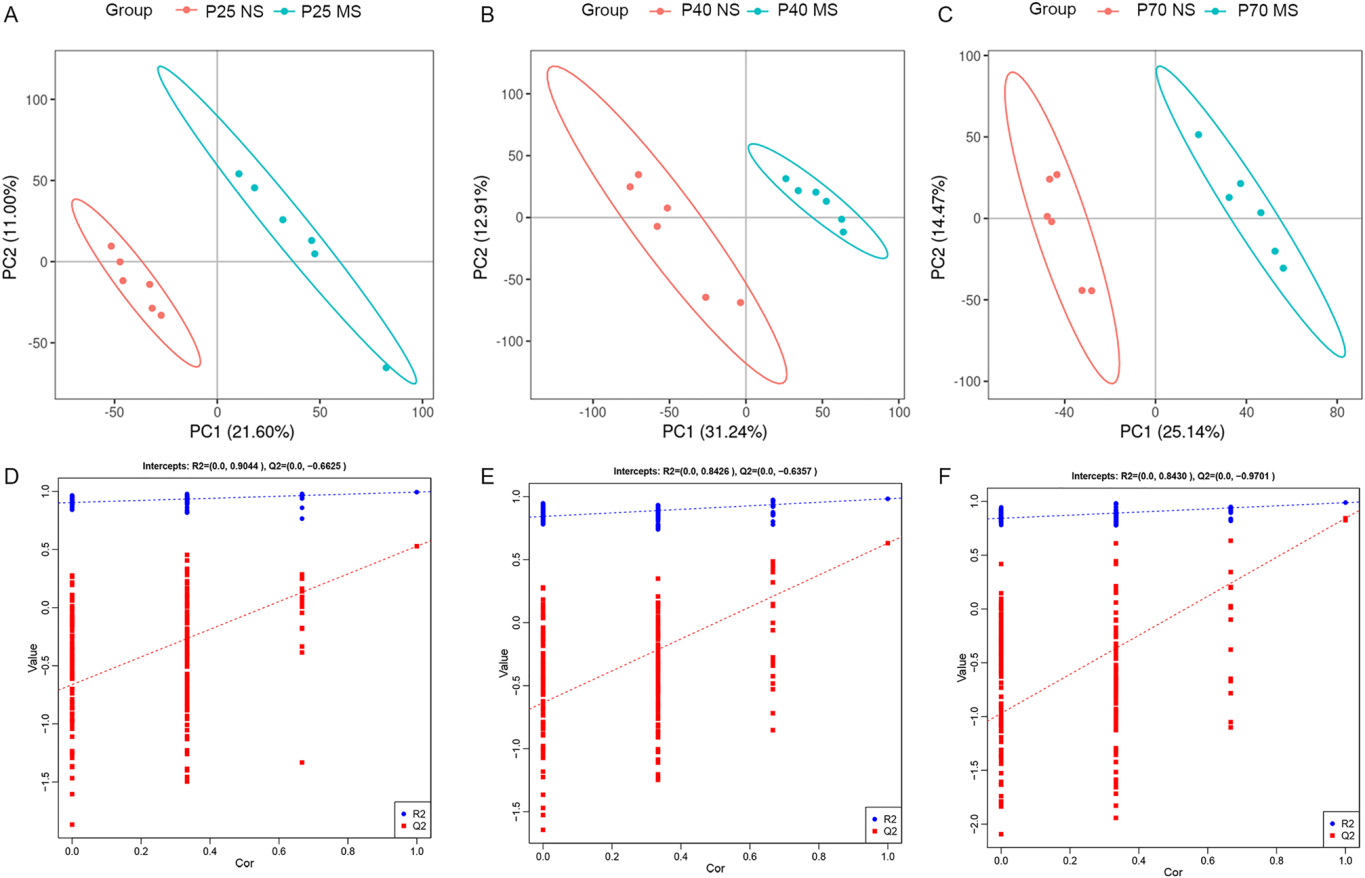
PLS-DA analysis model scores and test charts of MS and NS group in three age groups. (**A**)–(**C**) respectively showed the scores of PLS-DA analysis models of the two groups on P25, P40, and P70 days. (**D**)–(**F**) respectively represented the PLS-DA analysis model response ranking test diagrams on P25, P40, and P70. The two points on the right were the simulated real R2 and Q2 values, respectively, and the other points were the R2 and Q2 values obtained after random arrangement of the samples used. MS, maternal separation; NS, non-separation.

In this study, the supervised PLS-DA multivariate method was used to obtain the data of the first two main components of each group for reconstruction analysis. The results showed that the principal components of the metabolites in the two groups of three age groups were significantly different (Fig. 3A–C). In addition, 200 cross-verifications were performed on constructed model parameters R2 and Q2. The results showed that the R2 and Q2 values at the three time points were all greater than 0.5, indicating that the PLS-DA model had good quality and prediction ability (Fig. 3D–F).

### Comparison of different metabolites of ileal contents between MS group and NS group at different ages

**Figure 4:**
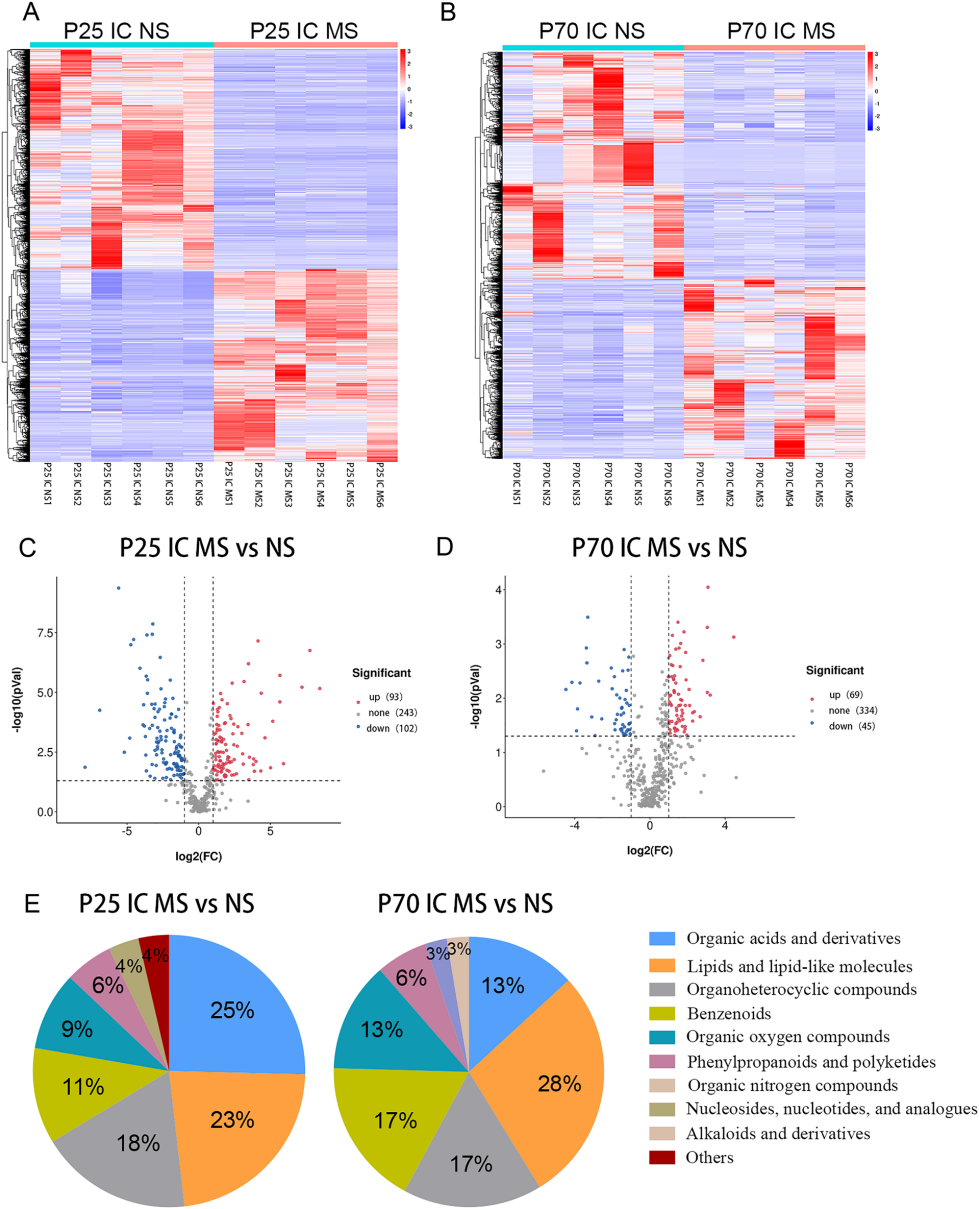
Comparison of different metabolites of ileal contents between MS group and NS group at different ages. (**A**) and (**B**) respectively showed the ion intensities of metabolites detected in the samples of ileal contents of the two groups on P25 and P70. (**C**) and (**D**) represented the metabolites detected in the two groups of ileal content samples on P25 and P70. (**E**) represented the classification composition of the differential metabolites detected in the ileal content samples of the two groups on P25 and P70. MS, maternal separation. NS, non-separation. IC, ileal contents.

The cluster heatmap of metabolite ions of ileal contents in the MS and NS groups at different ages was distinguishable, indicating that the ion intensity of metabolites in the two groups at different ages changed significantly (Fig. 4A,B). At P25, a total of 438 metabolites were detected in the ileal contents of the MS group compared to the NS group, of which 195 metabolites were different (93 were upregulated and 102 were downregulated) (Fig. 4C). A total of 448 metabolites were detected at P70, of which 114 were differentially expressed (69 were upregulated and 45 were downregulated) (Fig. 4D). At P25, the top four metabolites were organic acids and their derivatives (25%), lipids and lipid molecules (23%), organic heterocyclic compounds (18%), and benzene ring compounds (11%) (Fig. 4E). At P70, the top four metabolites were the same as for P25, although their proportions were different (Fig. 4E). Additionally, 58 metabolites were different between the two age groups, mainly lipids and lipids.

### Comparison of differential metabolites between fecal and ileal contents

**Figure 5:**
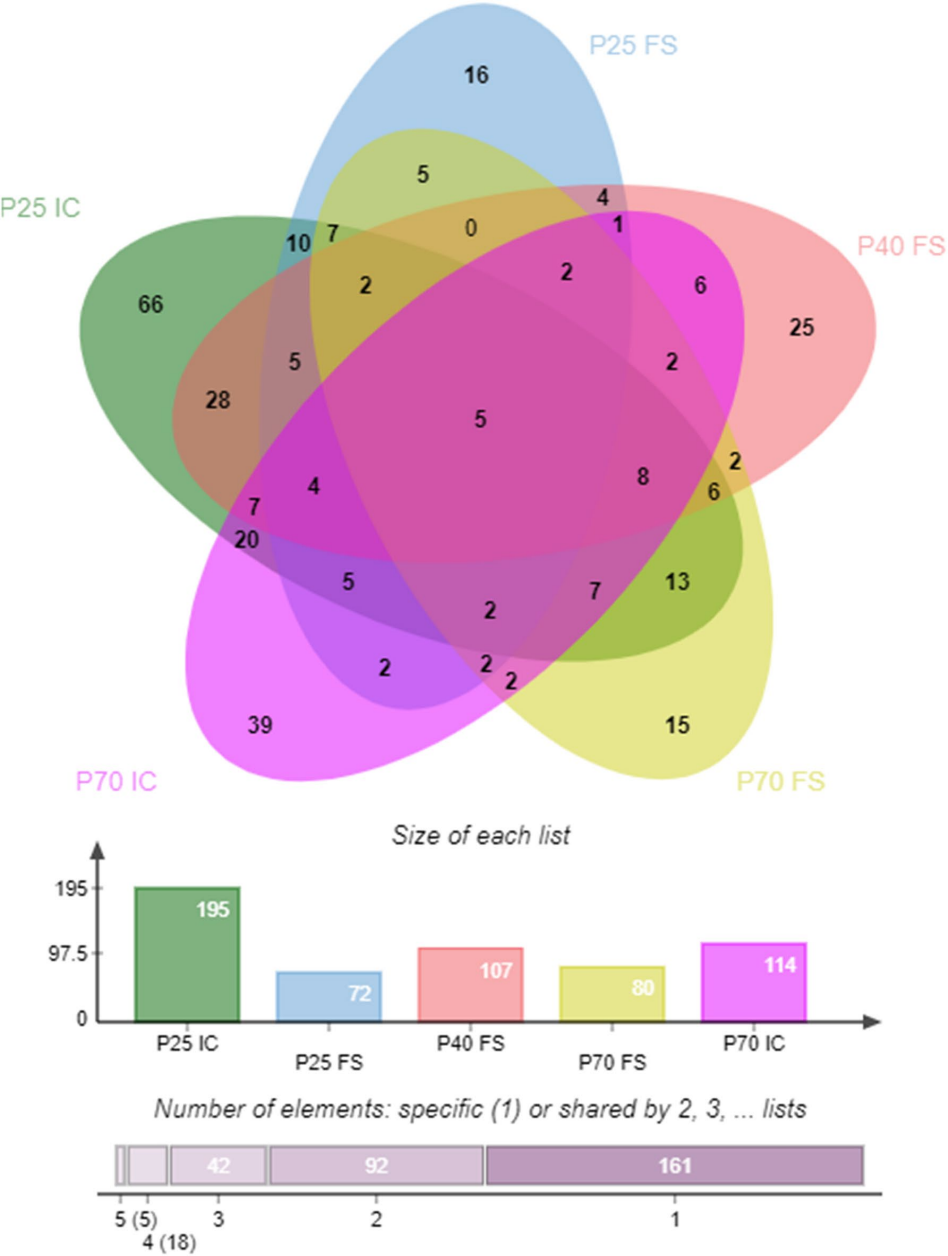
Wayne diagram of different metabolites detected in feces and ileal contents at different ages. FS, feces; IC, ileal contents.

Five identical differential metabolites were detected in the feces and ileal contents of the MS and NS groups at different ages, namely, benzamide, taurine, acetyl-L-carnitine, indole, and ethylbenzene (Fig. 5). We also found 20 different metabolites in the ileum contents that were not different in the feces, whereas there were no differences in the different metabolites for the feces. In addition, there were more differential metabolites in the ileal contents than in the fecal samples for each age group, and the classification of changes was also different.

### Metabolic pathway analysis

**Figure 6:**
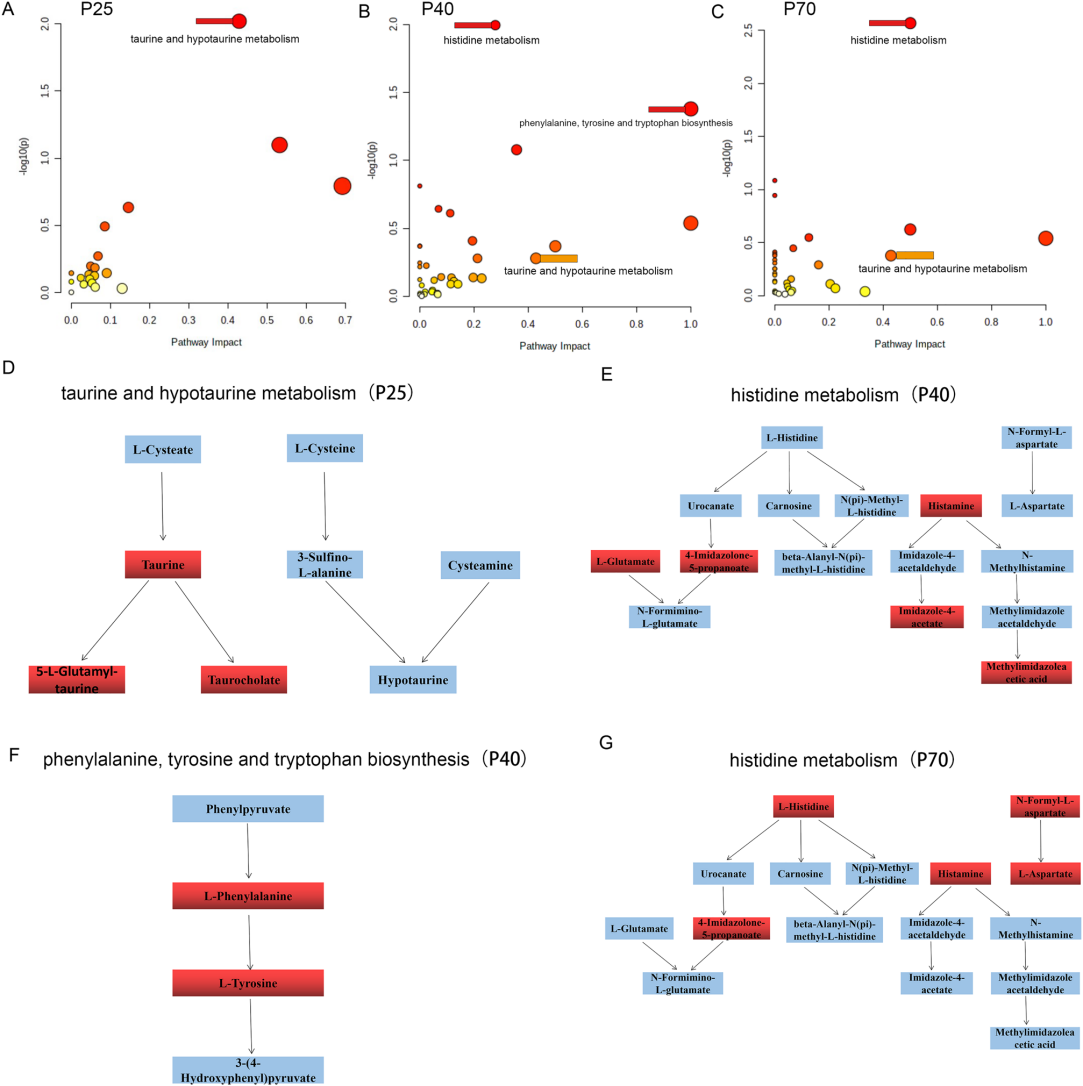
Changes of affected pathways in MS and NS group at different ages. (**A**), (**B**), and (**C**) represented the changes of the affected pathways on P25, P40, and P70 days. (**D**), (**E**), (**F**), and (**G**) indicated the names of the most affected pathways and the participating substances at different ages.

To further explore the influence of differential metabolites on metabolic pathway-related pathways, this study used MetaboAnalyst 5.0 to conduct comprehensive enrichment analysis and pathway analysis of the detected differential metabolites to identify the most relevant pathways. The results showed that taurine and hypotaurine metabolism were the most relevant pathways at P25, with an impact value of 0.428 (Fig. 6A,D); At P40, histidine metabolism was the most relevant pathway, with an impact value of 0.278. In addition, phenylalanine, tyrosine, and tryptophan biosynthesis were also affected, with impact values of 1.0 (Fig. 6B,E,F). Histidine metabolism was the most relevant pathway at P70, with an impact value of 0.499 (Fig. 6C,G), and was significantly affected at P40 and P70, while taurine and hypotaurine metabolism were affected in the three age groups with gradually decreasing influence.

## Discussion

Visceral hypersensitivity is a potentially important pathogenic factor in IBS patients. Visceral hypersensitivity is one of the main characteristics of IBS and has been widely studied in MS model rats^23,24^. Many animal experiments have shown that early MS can induce visceral hyperalgesia^7,25^. However, most experiments studied a single time point in childhood or adulthood so there was a lack of continuous dynamic impact studies. In particular, mice models of IBS are rarely used. Therefore, in this study, newborn mice with MS were used to construct an IBS model to induce visceral hypersensitivity, showing that MS-induced visceral hyperalgesia in IBS mice lasted from infancy to adulthood. In another study, Yi et al.^26^ reached similar conclusions using an IBS rat model, indicating that EALs induced visceral hyperalgesia may play an important role in IBS etiology.

In order to avoid the influence of the female physiological cycle on body endocrine, most of literature in this field used male mice^7,25^. However, another study observed that sex differences exist in the visceral hypersensitivity of MS and NS rats^26^. Meleine et al.^27^ reached sex ratio in IBS was highly skewed towards the female gender. Whether there is gender difference in visceral hypersensitivity remains to be further explored.

To the best of our knowledge, this study was the first to report the metabolomic characteristics of IBS mouse models in childhood, adolescence, and adulthood, and revealed that the ileal contents and fecal metabolites of the mice in the MS group were significantly different from those in the NS group. This difference did not recover due to MS termination, which lasted from childhood to adulthood.

On P25, the MS group showed a significant increase in kynurenine (Table S1), an important metabolite involved in the kynurenine pathway^28^. At the same time, kynurenine can be further metabolized as kynurenate. As a neuroprotective metabolite, kynurenate has been shown to activate the kynurenine pathway, leading to IBS^29^. As an antagonist of glutamic acid, kynurenic acid can inhibit the sensitivity of mucosal and vagus afferent mechanosensitivity, which played an important role in intestinal sensitivity^30^. Taurine and hypotaurine metabolism were the most relevant metabolic pathways in the P25 group. Taurine, an important substance in this pathway, was significantly downregulated in this age group. Taurine is a sulfur-containing amino acid that is not used for protein synthesis but exists as a free amino acid, which is endogenously generated by cysteine or methionine or exogenously provided by diet. Taurine was believed to regulate oxidative stress, osmotic pressure, intracellular calcium ion concentrations, and autophagy^31^. Taurine can also combine with bile acids to form bile, which may affect the biosynthesis of fatty acids, glycolysis, gluconeogenesis, and the metabolism of lipids and lipoproteins. Some studies have shown that taurine deficiency leaded to impaired bile acid metabolism, thus increasing the risk of similar mitochondrial diseases^32^. At the same time, taurine was involved in bile acid metabolism as a substrate for binding bile acid^33^, and many studies have shown that bile acid metabolism was involved in the pathological process of IBS^34–36^.

On P40, L-phenylalanine and L-tyrosine were significantly downregulated in the MS group (Table S1), and both were key metabolites in the biosynthesis pathway of phenylalanine, tyrosine, and tryptophan. Phenylalanine is an aromatic amino acid with lipophilic characteristics that affects the diffusion and transport of the membrane, changes the permeability of the membrane, and leads to mucosal barrier dysfunction. This may be one of the pathophysiological mechanisms of IBS^37^. Phenylalanine is a potential biomarker for IBS^38^. On P40 and P70, the levels of 4-imidazolone-5-propionate and histamine significantly decreased in the MS group, and both were important compounds in the histidine metabolism pathway. Histamine is a short-acting endogenous amine that is widely distributed in the human body, particularly in the skin, lungs, and digestive tract^39^. Histamine is involved in many physiological functions in the body, including cell proliferation, differentiation, regeneration, and regulation of the immune system^40^. At the same time, some studies have shown that histamine can change the movement of the gastrointestinal tract and the function of intestinal neurons, regulating the production of gastric acid and mucosal ion secretion^41–43^, indicating that changes in histamine levels were closely associated with IBS pathology. Studies have shown that receptor antagonists can be used to treat IBS, which can improve the symptoms of visceral hypersensitivity and abdominal discomfort^44^. Histamine also acts as a neurotransmitter in specific brain areas. Some studies have shown that decreased histamine levels in the brain can cause neurological symptoms such as anxiety in mice^45^. Previous studies have shown that long-term changes in neuroendocrine and neuropeptide secretions caused by MS may lead to visceral hyperalgesia such as corticotropin-releasing factor^46^ and nerve growth factor^47^. Therefore, whether histamine has the same effect as a neurotransmitter requires further verification. Some studies have shown that MS sensitizes the cingulate cortex and upregulates the activity of the ascending pathway at the spinal cord level and the thalamic cortex amygdala pathway to external stimuli, leading to visceral hypersensitivity^48^.

On P70, the linoleic acid level in the MS group was significantly downregulated (Table S1). Linoleic acid (LA) is an unsaturated fatty acid and a key precursor of conjugated linoleic acid (CLA). Previous studies have shown that CLA can regulate intestinal microbiota^49,50^ and inhibit anxiety, depression, and negative emotions^51,52^. Patients with IBS have a three-fold increased odds of either anxiety or depression^53^. The brain and intestine have a close two-way connection through the brain gut axis^54^. Negative emotions of the brain may be transmitted to the gut through the vagus nerve, causing visceral allergic reactions^55^. Simultaneously, stimulating the gut can cause the body to generate negative emotions^55,56^. Therefore, whether CLA can be used in IBS patients to improve intestinal visceral hypersensitivity and alleviate negative emotions is worth discussing in depth.

It is well known that gastrointestinal tract is an important place for the digestion, absorption, metabolism, and excretion of food, water, and nutrients. These activities of the gastrointestinal tract are subject to complex and precise regulation and are related to environmental conditions with location specificity^57^. Previous studies have shown that the physical and chemical compositions, water content, pH, microbiota, and intestinal contents of feces were also different^15,58,59^. Our study also confirmed that the metabolite compositions of feces and ileal contents were different. Due to the small number of ileal contents in the MS group on P40, no enough samples were taken for examination to reduce deviation. The differential metabolites of ileal contents in the other two periods were higher than those in the feces, and the main components were also different. Among these, the proportion of lipids and lipid molecules in ileal contents was lower than that of feces, whereas the proportion of organic acids and their derivatives was higher than that of feces.

Among the different metabolites in different parts of the three periods, five different metabolites were found to be shared by the ileum contents and feces, indicating that MS caused metabolite changes in these two parts which persisted from childhood to adulthood. Indole is synthesized, modified, degraded, or metabolized by tryptophan in many bacterial species through tryptophan enzyme^60^. The main function of indole is to act as an intercellular signaling molecule, enhancing the resistance of epithelial cells, inducing the expression of intestinal tight junction proteins, and reducing inflammatory cytokines^61,62^. Studies have shown that indole and indole derivatives played beneficial roles in establishing epithelial barriers and preventing intestinal inflammation^63,64^. Acetyl-l-carnitine is a derivative of lysine and methionine. It plays a role in energy metabolism, antioxidation, and regulation of brain neurotransmitters (acetylcholine, serotonin, dopamine, and so on)^65^. In addition, animal experiments on chronic pain showed that acetyl-l-carnitine can induce neuroprotection, neurotrophic, and analgesia^66,67^. Whether acetyl-L-carnitine also plays the same role in IBS requires further research and verification.

In conclusion, this study found that changes in differential metabolites and metabolic pathways in IBS models persisted from childhood to adulthood using non-targeted metabolomics, providing new insights into the etiology of IBS. However, this study had some limitations. The differential metabolites that were not targeted for detection have not been verified, and the altered metabolic pathways have not been jointly verified with other omics. This is also a direction for future research. In combination with other omics techniques, we aim to identify relevant biomarkers and provide therapeutic targets for IBS.

## Supplementary Information

Supplementary Table S1.

## Data Availability

The original contributions presented in this study are included in the article/Supplementary material, further inquiries can be directed to the corresponding authors.
