# Supplementary material for: Maternal separation leads to dynamic changes of visceral hypersensitivity and fecal metabolomics from childhood to adulthood
Source: Sci Rep. 2023 May 11;13:7670. doi: 10.1038/s41598-023-34792-7 (PMC10175246; doi:10.1038/s41598-023-34792-7)
Supplement: Supplementary file 1 — Supplementary Table S1. [file 41598_2023_34792_MOESM1_ESM.pdf]

Table S1. Different metabolites and pathway information of feces and ileum contents in MS and NS groups at three time points

Metabolite	RT	*P* value	Fold Change	VIP	Regulated	KEGG
**P25 FS**						
l-kynurenine	3.787	0.007658129	2.701609419	3.754014011	up	C00328
lysosm(d18:1)	7.016	0.024855994	110.1979409	3.340477483	up	C03640
3-dehydroteasterone	4.577	0.005954277	0.233180792	2.420099285	down	C15792
saccharopine	0.877	0.04512254	4.154979311	2.294679768	up	C00449
alpha-tocotrienol	5.173	0.034802037	0.175737664	2.286983027	down	C14153
3-hydroxyanthranilic acid	3.334	0.003193767	0.288640469	2.283729176	down	C00632
taurine	0.885	0.000102713	0.069051883	2.266744161	down	C00245
i-urobilin	4.536	0.019097125	0.287402422	2.188804496	down	C05794
dopamine	2.448	0.02973378	3.935012975	2.187986881	up	C03758
n-succinyl-2-l-amino-6-oxoheptanedioate	3.351	0.014777285	4.339013104	2.180516205	up	C04462
costunolide	4.568	0.011986069	0.333977987	2.164822341	down	C09382
20alpha-hydroxy-4-pregnen-3-one	6.425	0.02373206	0.22014731	2.133557345	down	C04042
styrene	4.563	0.041887733	0.406653858	2.133458786	down	C07083
2-hydroxyethanesulfonate	0.919	0.047053421	0.207830172	2.12785771	down	C05123
lysopc(22:5(7z,10z,13z,16z,19z))	5.681	0.024018557	3.358640846	2.072662508	up	C04230
daidzein	4.131	0.038787472	9.32358427	2.068195618	up	C10208
gamma-tocotrienol	6.151	0.039295035	0.168978771	2.055990562	down	C14155
cytosine	0.903	0.013609261	0.406183799	2.003336282	down	C00380
biphenyl	4.557	0.017403601	0.315366898	1.984092576	down	C06588
cinnamyl alcohol	5.615	0.018927739	0.485511387	1.978433482	down	C02394
chenodeoxyglycocholate	4.923	0.008766016	0.288445866	1.954240793	down	C05466
ferulic acid	3.543	0.035284757	14.02716258	1.922885995	up	C01494
cafeine	0.894	0.028046588	3.267741049	1.918635565	up	C07481
5-l-glutamyl-taurine	0.908	0.000659084	0.207734807	1.915367524	down	C05844
3alpha-hydroxy-5beta-cholanate	6.859	0.005483354	0.400339441	1.885663799	down	C03990
fexofenadine	5.588	0.011792728	3.242380586	1.869478769	up	C06999
porphobilinogen	3.357	0.042088052	7.427463308	1.847859799	up	C00931
6-hydroxynicotinic acid	2.273	0.042590714	4.333236351	1.801271161	up	C01020
adenine	0.985	0.045152712	3.168763177	1.775482328	up	C00147
5-methoxyindoleacetate	3.657	0.03756091	2.293842814	1.772663983	up	C05660
kahweol	4.605	0.000673528	0.319689876	1.73441373	down	C09893
s-lactoylglutathione	4.545	0.043931272	0.346902999	1.726943978	down	C03451
luteolin	4.145	0.034631616	10.11481754	1.713249394	up	C01514
4-(methylnitrosamino)-1-(3-pyridyl-n-oxide)-1-butanol	3.208	0.020926276	2.796435353	1.70276149	up	C19603
l-cystine	0.858	0.043110519	4.148735589	1.693378983	up	C00491
5-oxoete	5.054	0.042834562	0.385505165	1.675276893	down	C14732
ethylbenzene	5.618	0.037712536	0.499082122	1.661308417	down	C07111
acetyl-l-carnitine	3.716	0.012317953	3.656125938	1.656739911	up	C02571
sn-glycero-3-phosphoethanolamine	0.869	0.00809471	2.921334173	1.611081513	up	C01233
8-amino-7-oxononanoate	4.013	0.02224047	2.400001931	1.582795833	up	C01092
s-(hydroxymethyl)glutathio	0.962	0.049673515	3.059673496	1.575374494	up	C14180

ne						
ecgonine	3.852	0.038384289	2.55451922	1.558209512	up	C10858
(r)-prunasin	3.765	0.037769576	3.93729646	1.53298631	up	C00844
lysopc(20:5(5z,8z,11z,14z,17z))	5.263	0.045895591	2.236079131	1.524528818	up	C04230
oxytetracycline	0.867	0.001643319	0.119072291	1.51894749	down	C06624
7-hydroxy-6-methyl-8-ribityllumazine	0.937	0.023953843	3.021142272	1.502378566	up	C05995
1-palmitoylglycerophosphocholine	5.784	0.047584831	2.080736921	1.487411297	up	C04230
6-hydroxymelatonin	3.685	0.035779455	0.323787436	1.482889424	down	C05643
linatine	0.983	0.009924066	2.399192756	1.460086784	up	C05939
n-acetyl-l-citrulline	0.889	0.013230887	2.082278864	1.40646178	up	C15532
cortolone	4.325	0.024043635	0.247938359	1.398938812	down	C05481
d-sphingosine	4.964	0.00976351	0.417451355	1.3732426	down	C00319
sulfate	1.058	0.004115355	0.338703299	1.372570431	down	C00059
dhurrin	3.615	0.036765942	2.692626355	1.371987025	up	C05143
5'-s-methyl-5'-thioadenosine	3.433	0.022755799	2.899897704	1.370588457	up	C00170
deoxycholic acid	5.09	0.019014672	0.452173174	1.360601263	down	C04483
orthophosphate	0.908	0.036239738	4.989788533	1.359026304	up	C00009
l-threonic acid	0.943	0.041755655	3.005314717	1.347540089	up	C01620
uric acid	0.982	0.008311551	0.437395566	1.312261181	down	C00366
indole	5.62	0.046800132	0.49973662	1.309425587	down	C00463
glycochenodeoxycholic acid	4.925	0.005439661	0.445405017	1.303478454	down	C05466
benzamide	4.302	0.029891079	3.100268287	1.302703575	up	C09815
methylimidazoleacetaldehyde	3.377	0.017295506	0.45359601	1.298050157	down	C05827
2-keto-4-methylthiobutyric acid	3.399	0.047668	0.170155571	1.297385866	down	C01180
jasmonic acid	5.644	0.009270293	3.240744797	1.271237109	up	C08491
5beta-cyprinolsulfate	4.904	0.01368171	0.202564268	1.183141302	down	C05468
taurocholic acid	4.091	0.011240613	0.082517717	1.175346894	down	C05122
trans-4-hydroxy-l-proline	3.361	0.034303193	2.548201222	1.154335656	up	C01157
sulfite	0.915	0.004720211	0.32488479	1.069793789	down	C00094
paraxanthine	0.98	0.015187197	2.634403541	1.048668894	up	C13747
3-hydroxyphenylacetic acid	3.574	0.013590855	0.482715731	1.029337178	down	C05593
citric acid	0.991	0.004084747	2.338891072	1.02119012	up	C00158
**P25 IC**						
porphobilinogen	3.598	6.10E-06	147.5762783	3.082987952	up	C00931
indole	3.465	4.28E-10	0.020803516	2.89594661	down	C00463
atrazine-desethyl	3.53	1.77E-07	215.7411395	2.797258045	up	C06559
coumarin	1.039	0.000257694	0.072414585	2.729827209	down	C05851
toluene	3.273	0.000711389	0.160701576	2.715317839	down	C01455
l-citrulline	0.888	6.12E-08	0.042981247	2.682500874	down	C00327
imidazolone	1.077	1.94E-06	50.73454523	2.569161445	up	C06195
benzamide	1.617	0.00267613	0.120806217	2.495727853	down	C09815
oxytetracycline	0.867	5.63E-05	0.008352588	2.458951752	down	C06624
l-formylkynurenine	2.116	0.000836828	0.132695302	2.428802729	down	C02700
d-(+)-maltose	0.899	0.000799462	24.64242828	2.381278813	up	C00208
2-hydroxy-3-	0.905	0.000118655	0.251006452	2.378987137	down	C01146

oxopropanoate						
5-l-glutamyl-taurine	0.908	9.76E-07	0.058518417	2.339098771	down	C05844
4-anisic acid	3.5	0.001795006	6.436932497	2.30337231	up	C02519
arachidonic acid	7.852	1.90E-05	0.201114612	2.296370817	down	C00219
l-tyrosine	1.686	0.001872563	0.136314266	2.293131775	down	C00082
felbamate	3.366	0.009680982	60.18625501	2.279324501	up	C07501
benzaldehyde	3.274	0.012870709	0.331555056	2.220167598	down	C00261
berberine	4.099	0.006488571	5.553356542	2.215158537	up	C00757
piperidine	2.015	2.10E-06	0.079056822	2.20937414	down	C01746
adenosine	3.279	2.51E-05	50.6547866	2.207895927	up	C00212
8-methoxykynurenic acid	3.799	6.89E-06	348.605224	2.164917832	up	C05830
parathion	0.787	2.99E-06	0.084564891	2.139824112	down	C06604
2-oxoglutaric acid	1.462	0.004226024	0.077423773	2.130683764	down	C00026
l-kynurenine	3.226	0.005244236	4.294793841	2.093243833	up	C00328
2'-deoxyinosine	0.987	6.35E-07	11.10196262	2.035171508	up	C05512
n-arachidonoylethanolamine	4.208	0.006108367	0.182998418	2.007794283	down	C11695
glycitein	4.178	0.000111587	0.121015865	1.964300238	down	C14536
paraxanthine	1.67	4.82E-05	0.115802793	1.95355755	down	C13747
sulfite	0.915	7.03E-08	17.55383463	1.939107367	up	C00094
sn-glycero-3-phosphoethanolamine	0.884	0.000212893	5.458330371	1.934035312	up	C01233
2-oxoglutaramate	1.02	0.011542063	0.342119271	1.934022185	down	C00940
l-alanine	0.87	0.000296749	0.138019512	1.927847362	down	C00099
5-hydroxy-n-formylkynurenine	0.984	0.029010132	4.933911529	1.919312643	up	C05648
cuminaldehyde	8.461	0.002666055	3.821203659	1.89207022	up	C06577
5-methylcytosine	0.9	0.003745952	8.360488789	1.89112741	up	C02376
adenine	0.994	2.98E-05	3.989388637	1.884153115	up	C00147
thymidine	3.278	0.001138172	0.145142803	1.841704683	down	C00214
5-phosphoribosylamine	3.476	3.96E-08	0.081726371	1.827652536	down	C03090
l-isoleucine	3.683	0.001561461	5.474370034	1.827002106	up	C00407
skatole	3.468	0.001070568	0.2039815	1.824868723	down	C08313
narcotoline	3.764	0.001317647	0.139904787	1.822492791	down	C09593
chenodeoxyglycocholate	4.969	1.01E-07	0.037495103	1.800003993	down	C05466
acetyl-l-carnitine	1.52	0.000187493	3.614899039	1.784903797	up	C02571
l-valine	1.011	0.00070644	0.076134023	1.782477757	down	C00183
l-methionine sulfoxide	0.914	0.00048382	0.306467069	1.774244485	down	C02989
5-aminolevulinic acid	1.625	0.005043916	4.097665893	1.766038778	up	C00430
8z,11z,14z-eicosatrienoic acid	8.229	0.018034565	0.35600748	1.763275077	down	C03242
1-methylxanthine	0.938	0.001879517	0.344519514	1.749704856	down	C16358
6-hydroxymelatonin	3.685	0.020082855	14.77534065	1.746776227	up	C05643
hippuric acid	3.765	0.000521469	8.025375615	1.745798467	up	C01586
n1-methyl-2-pyridone-5-carboxamide	0.982	0.000124908	4.778304216	1.743086678	up	C05842
dihydrozeatin-o-glucoside	1.663	0.032290506	0.095784581	1.738022014	down	C16448
cortolone	4.325	3.07E-05	0.121738991	1.732889298	down	C05481
3-methoxy-4-hydroxyphenylethyleneglycol	4.014	0.009090121	3.811435306	1.731071208	up	C05594

[6]-gingerol	4.453	0.003414734	0.168848736	1.727276627	down	C10462
4-(nitrosoamino)-1-(3-pyridinyl)-1-butanone	0.999	2.03E-05	3.087036213	1.693338043	up	C19564
xanthine	1.008	6.45E-05	0.139748502	1.676951587	down	C00385
l-glutamic acid	0.884	0.003124554	0.322808969	1.660695152	down	C00025
chenodeoxycholic acid	5.492	0.000822187	0.035735825	1.658186153	down	C02528
daidzein	4.138	0.009484855	0.206385421	1.656074293	down	C10208
2-aminoacrylate	0.886	0.000448931	0.297868688	1.65358396	down	C02218
physostigmine	3.347	0.000228763	0.067743146	1.643523976	down	C06535
betaine	0.922	0.000273303	6.652448131	1.64282019	up	C00719
estriol	3.401	0.002251968	0.339146809	1.633571087	down	C05141
phenol	1.035	0.014125857	0.38117019	1.630899764	down	C00146
docosahexaenoic acid	7.694	0.014080695	0.475105686	1.630739249	down	C06429
salsolinol	4.074	0.010669973	2.601956278	1.617879674	up	C09642
n-acetylneuraminic acid	0.968	3.31E-05	0.06966865	1.608492942	down	C00270
capecitabine	3.427	2.72E-05	0.19414743	1.601022487	down	C12650
1-aminocyclopropane-1-carboxylate	0.899	0.000628461	0.41994383	1.598182765	down	C01234
d-serine	0.879	5.11E-05	0.150093628	1.597911745	down	C00740
2-hydroxyethanesulfonate	0.919	3.07E-06	0.253597865	1.595070088	down	C05123
acrylic acid	0.946	5.16E-05	0.377748311	1.580139721	down	C00511
4-acetamido-2-amino-6-nitrotoluene	3.263	3.69E-08	0.105805772	1.56946211	down	C16420
6-hydroxypseudooxynicotine	3.456	9.41E-05	4.536493282	1.552097086	up	C01297
androsterone	4.783	0.002332608	0.135962534	1.54434304	down	C00523
n-acetylornithine	0.916	0.018550026	0.386822465	1.544212793	down	C00437
3-sulfinylpyruvate	0.933	0.006985264	0.441855462	1.537837497	down	C05527
genistin	3.562	1.09E-05	20.5730863	1.535416058	up	C09126
propynoate	0.934	0.001537425	0.38703513	1.534655141	down	C00804
histamine	0.997	1.11E-05	2.890282965	1.523292721	up	C00388
coniferylaldehyde	7.826	0.018211965	3.917505221	1.522363259	up	C02666
5-hydroxyindole-3-acetic acid	3.481	0.007796889	15.9274027	1.507489048	up	C05635
isoliquiritigenin	4.16	0.000240106	0.105765596	1.506736259	down	C08650
l-cystine	0.864	0.003216603	0.027409024	1.50669777	down	C00491
inosine	2.073	0.000223801	10.83995892	1.503721165	up	C00294
gossypol	0.937	0.010042653	8.60604748	1.500529895	up	C07667
4-methylene-l-glutamate	0.934	0.000100923	2.446204217	1.491917591	up	C00651
phloionolic acid	4.352	0.031099183	3.440922612	1.485861087	up	C19621
1-pyrroline-4-hydroxy-2-carboxylate	4.064	3.52E-06	8.935245534	1.481086473	up	C04282
5beta-cyprinolsulfate	4.53	0.045194917	11.22807925	1.474100467	up	C05468
20alpha-hydroxy-4-pregnen-3-one	7.658	0.012305752	2.471823372	1.457331318	up	C04042
3-methyldioxyindole	3.604	1.09E-05	0.083803379	1.456137794	down	C05834
homovanillic acid	3.591	1.37E-08	0.10885405	1.455147272	down	C05582
allicin	0.986	0.028428135	3.533045847	1.449018784	up	C07600
octanoylglucuronide	3.688	0.028229777	0.357112912	1.446348027	down	C03033
cyclohexanecarboxylic acid	6.764	0.021750459	2.942002398	1.44500087	up	C09822
pilocarpine	4.298	0.019577927	20.14052385	1.435006634	up	C07474

norepinephrine	3.439	0.006128396	0.429867575	1.431235162	down	C00547
m-trehalose	0.863	0.000162106	35.9652632	1.425015748	up	C01083
melatonin	3.564	0.00862091	4.501434207	1.411172398	up	C01598
4-imidazolone-5-propanoate	0.933	0.003267054	3.276721434	1.40247021	up	C03680
1-palmitoylglycerophosphocholine	7.36	0.036690456	0.209154004	1.397313545	down	C04230
2-hydroxycinnamic acid	3.876	3.44E-07	0.155544828	1.394787962	down	C01772
imidazoleacetic acid	1.467	0.012490987	2.931394215	1.366858495	up	C02835
l-aspartic acid	0.876	0.000944471	0.16309734	1.366766966	down	C00049
saccharopine	1.494	0.038962135	0.438429401	1.358897514	down	C00449
methylimidazoleacetaldehyde	0.917	0.000396534	2.451582342	1.355707894	up	C05827
citric acid	0.991	0.008576982	0.182615212	1.348628916	down	C00158
naringenin	4.291	0.039810741	0.119874883	1.347886412	down	C00509
d-raffinose	0.904	0.008002751	4.882742435	1.34573382	up	C00492
3-hydroxybutyric acid	1.984	0.032731499	3.721919582	1.344471199	up	C01089
thymine	3.281	0.039698053	0.176756307	1.344134384	down	C00178
1-nitrosonaphthalene	1.757	0.000383191	0.306061354	1.341743128	down	C14788
citraconic acid	1.01	0.027326029	0.41228832	1.333082569	down	C02226
morphinone	4.083	0.006411443	4.033720122	1.328921441	up	C01735
l-glutamine	3.825	0.001188543	0.130466114	1.325929801	down	C00064
lysopc(p-18:0)	7.359	0.000143033	0.192450757	1.317718436	down	C04230
dopamine	0.926	0.000336547	2.459656093	1.313872049	up	C03758
6-hydroxynicotinic acid	2.273	0.016484706	0.272572987	1.312018796	down	C01020
n-formyl-l-aspartate	0.954	0.045580747	2.505453976	1.308116288	up	C01044
orthophosphate	5.1	0.000201227	3.463248473	1.299630565	up	C00009
8(s)-hydroxy-(5z,9e,11z,14z)-eicosatetraenoic acid	5.901	3.97E-05	0.189866397	1.295830323	down	C14776
cholic acid	4.813	0.000322907	0.093570089	1.294898396	down	C00695
biliverdin	4.716	0.028446446	2.386066176	1.292050905	up	C00500
glutamic acid	9.854	6.51E-05	2.522659349	1.279390959	up	C00025
naphthalene	4.817	0.011446851	0.128088445	1.27252188	down	C00829
11-cis-retinol	9.652	0.000923465	3.318374452	1.271181008	up	C00899
chavicol	5.211	0.030108374	3.703487176	1.270894448	up	C16930
5-oxoete	5.408	0.015206737	0.242187582	1.264731103	down	C14732
guanine	0.99	0.047116695	2.250549008	1.259583051	up	C00242
(s)-2-acetolactate	0.909	0.025312825	0.498532619	1.253605794	down	C06010
beta-aminopropionitrile	9.798	0.001082974	3.020934822	1.251899696	up	C05670
glycine	0.865	0.00409	0.256784591	1.237406869	down	C00037
l-(-)-methionine	3.831	0.012216494	0.364928601	1.231718813	down	C00073
n6,n6,n6-trimethyl-l-lysine	0.987	7.13E-06	0.161136493	1.229438204	down	C03793
lithocholic acid	8.301	0.006527993	2.285914146	1.228007262	up	C03990
palmitoylcarnitine	5.222	0.005826237	0.444685495	1.226903428	down	C02990
phenylglyoxylic acid	3.561	0.049482472	2.947836458	1.220024698	up	C02137
mivacurium	4.338	0.014974729	3.264051191	1.218965857	up	C07550
taurine	4.343	0.014417244	32.23864458	1.215333205	up	C00245
(s)-1-pyrroline-5-carboxylate	1.013	0.00142344	2.325334429	1.213780223	up	C03912
estrone	3.447	0.045217131	0.257098151	1.210065154	down	C00468

4-hydroxybenzaldehyde	3.568	0.010217658	3.427791997	1.202230729	up	C00633
pantothenol	3.384	0.007368233	2.873433997	1.193335842	up	C05944
d-glucosaminide	4.564	0.010116605	0.201508581	1.192051693	down	C06023
3-ureidopropionic acid	0.855	3.16E-05	0.1035265	1.190670542	down	C02642
pregnenolone	6.006	0.011318316	3.24122242	1.185191963	up	C01953
ascorbic acid	4.521	0.019422591	0.079616966	1.184594227	down	C00072
2-phosphoglycolate	9.918	0.00109011	2.634535861	1.18308803	up	C00988
pyruvic acid	1.019	0.003621981	0.310022497	1.182520876	down	C00022
gentisic acid	3.685	0.009718617	3.225812638	1.182146617	up	C00628
(-)-trans-carveol	7.933	0.000241403	2.631051597	1.178791627	up	C00964
capsidiol	4.287	0.009827782	0.301357544	1.176448235	down	C09627
mesaconic acid	0.951	0.02705648	0.464017836	1.176367525	down	C01732
d-(+)-malic acid	1.115	0.000723436	0.206554635	1.17006405	down	C00497
butanal	1.82	0.007343878	2.777382731	1.168105685	up	C01412
3alpha-hydroxy-5beta-cholanate	8.483	0.003056568	2.511332708	1.167325291	up	C03990
ferulic acid	3.543	4.12E-06	5.045917086	1.164627326	up	C01494
2-methyl-3-hydroxy-5-formylpyridine-4-carboxylate	3.54	0.007066752	0.440174912	1.156655738	down	C06050
kynurenic acid	3.93	0.00420247	0.382447725	1.155132941	down	C01717
sulfate	4.501	0.013754785	2.729116341	1.153470984	up	C00059
xanthurenic acid	3.638	8.17E-05	0.269874932	1.150845809	down	C02470
尾-muricholic acid	4.821	0.000420999	0.153093606	1.142603034	down	C17726
p-cymene	7.452	0.020786852	2.504327409	1.136514976	up	C06575
pantothenic acid	3.264	0.004433381	0.383704227	1.129409829	down	C00864
n-(4-guanidinobutyl)-4-hydroxycinnamide	3.491	0.001873743	0.414946378	1.128836052	down	C04498
n-carbamoylputrescine	0.894	8.04E-05	0.267401474	1.128735783	down	C00436
ethylbenzene	4.816	0.004115904	0.130152258	1.124611494	down	C07111
8-amino-7-oxononanoate	6.907	0.015738578	3.030978273	1.123171285	up	C01092
leukotriene d4	7.686	0.000220842	2.382847091	1.12162444	up	C05951
palmitoleic acid	5.907	4.36E-05	2.706685426	1.119018141	up	C08362
jasmonic acid	5.49	0.011931731	2.09245457	1.112377041	up	C08491
lysopc(22:4(7z,10z,13z,16z))	6.128	0.001521349	3.673437873	1.097489335	up	C04230
deoxycholic acid	5.496	0.013666512	0.004105678	1.096767743	down	C04483
5-alpha-thdoc	4.321	0.002534178	0.168706462	1.092215284	down	C13713
udp-n-acetyl-d-mannosamine	0.927	0.025663461	6.220711115	1.089276075	up	C01170
threonine	0.879	0.018968862	0.422241074	1.07786395	down	C00188
cytosine	3.188	0.001199582	3.028999888	1.069261176	up	C00380
4-(methylnitrosamino)-1-(3-pyridyl-n-oxide)-1-butanol	0.981	0.016135896	2.005108638	1.068215768	up	C19603
taurochenodeoxycholic acid	4.789	0.005214133	11.62835442	1.063857933	up	C05465
xanthosine	3.249	0.000320081	0.269422065	1.057221612	down	C01762
l-phenylalanine	3.931	0.0129062	0.441533637	1.046566489	down	C00079
costunolide	6.559	0.00322139	2.650212576	1.041578014	up	C09382
caffeine	4.152	0.007907246	4.335916802	1.035872657	up	C07481
4-acetamidobutanoic acid	3.522	0.004933628	2.700442862	1.032613077	up	C02946
biphenyl	4.557	0.009545115	0.302129103	1.032144972	down	C06588

(-)-beta-pinene	5.132	0.000823185	2.971923814	1.030677556	up	C06307
caprolactam	3.638	0.001024381	2.491241834	1.02326544	up	C06593
3-hydroxyanthranilic acid	3.334	0.019705277	3.014641873	1.01981591	up	C00632
n-acetylputrescine	0.974	0.019978425	4.344562166	1.018164384	up	C02714
rutin	9.308	0.000369938	2.790208975	1.018082579	up	C05625
10-hydroxycarbazepine	4.516	0.001971452	0.440235783	1.011222034	down	C07493
l-histidine	0.981	0.000566603	2.094313149	1.003995254	up	C00135
**P40 FS**						
saccharopine	0.877	8.48E-05	0.220549181	3.046771567	down	C00449
morphinone	4.083	0.046954069	0.090099826	3.022778407	down	C01735
1-aminocyclopropane-1-carboxylate	1.925	0.002547531	0.030586111	2.859349349	down	C01234
imidazoleacetic acid	1.467	0.00221187	0.129224641	2.782946835	down	C02835
l-isoleucine	3.38	0.028722601	0.195271088	2.7747609	down	C00407
cytosine	3.188	0.00064902	0.113358134	2.706858545	down	C00380
udp-n-acetyl-d-mannosamine	0.927	0.000127182	11.2670131	2.603962435	up	C01170
alpha-tocotrienol	9.287	0.007920806	3.852416145	2.583449796	up	C14153
cortol	5.742	0.007623055	4.593221457	2.553589106	up	C05482
toluene	3.959	0.000203865	0.207101094	2.522564224	down	C01455
3-hydroxyanthranilic acid	3.334	4.70E-07	4.988162789	2.499039847	up	C00632
4-(methylnitrosamino)-1-(3-pyridyl-n-oxide)-1-butanone	0.994	0.00019741	0.357673341	2.410764805	down	C19602
2-hydroxy-3-(4-hydroxyphenyl)propenoate	3.408	0.001817752	0.194516389	2.378217781	down	C05350
xanthosine	3.249	0.03034622	0.034265725	2.340566385	down	C01762
pantothenic acid	3.375	0.002317539	0.333079914	2.330898191	down	C00864
jasmonic acid	5.644	0.04508082	17.83245107	2.280627422	up	C08491
2,2-dichloro-1,1-ethanediol	1.624	0.000481231	0.126708178	2.238436091	down	C14860
lysopc(22:4(7z,10z,13z,16z))	6.128	0.015994158	11.46817199	2.187873434	up	C04230
octanoylglucuronide	3.688	0.000480923	4.482811025	2.171593098	up	C03033
npc	3.969	0.020208545	0.21909516	2.139591666	down	C16543
histamine	0.804	0.002941943	6.920323537	2.074575063	up	C00388
salicylic acid	3.661	0.012811504	3.011269286	2.069385551	up	C00805
estriol	3.492	0.004418438	0.413639787	2.045163586	down	C05141
4-imidazolone-5-propanoate	0.933	0.000218946	0.316863897	1.99689779	down	C03680
hippuric acid	3.765	0.02047486	4.189067697	1.978650925	up	C01586
ascorbic acid	3.294	0.028737918	0.006101145	1.966480599	down	C00072
d-arabinonate	0.911	0.00940438	0.283426532	1.947211167	down	C00878
4-(beta-acetylaminoethyl)imidazole	3.383	0.027474424	0.384521321	1.926464008	down	C05135
zalcitabine	1.751	0.000981189	0.423437573	1.916901007	down	C07207
melatonin	5.051	0.018998917	0.314110212	1.898058151	down	C01598
5-aminopentanamide	0.928	0.009681816	0.085172291	1.885902467	down	C00990
n1-methyl-2-pyridone-5-carboxamide	2.173	0.006790399	0.363169688	1.862642231	down	C05842
cytidine	1.471	0.002639503	0.347071012	1.855547365	down	C00475
threonine	0.879	0.009670422	0.378339727	1.852042732	down	C00188
n-acetyl-l-phenylalanine	3.835	0.013729683	0.129809169	1.829194593	down	C03519

2-aminomuconate	0.953	0.005169063	0.271027953	1.828073297	down	C02220
2-oxoglutaramate	1.02	0.007682087	0.424960276	1.821966934	down	C00940
l-glutamic acid	0.884	0.011249633	0.440112653	1.813811881	down	C00025
l-phenylalanine	3.358	0.004505931	0.198594979	1.812978099	down	C00079
[6]-gingerol	4.453	0.02960037	7.185489081	1.808346216	up	C10462
guanosine	2.054	0.004324947	0.265933452	1.803356529	down	C00387
lysopc(22:5(7z,10z,13z,16z,19z))	5.681	0.044725043	7.460645925	1.800915267	up	C04230
hypoxanthine	3.31	0.023576319	0.120455442	1.800287665	down	C00262
adenosine	3.279	0.014774835	0.068602366	1.797610608	down	C00212
styrene	7.161	0.003924238	3.225841273	1.781641554	up	C07083
sulfosalicylic acid	3.41	0.002380722	10.14662679	1.758747283	up	C16199
agmatine	0.81	0.039046672	2.196254862	1.756785295	up	C00179
gamma-l-glutamylputrescine	0.834	0.037349135	0.281183672	1.744856181	down	C15699
ferulic acid	3.543	0.018209794	0.311208018	1.725668057	down	C01494
ethylbenzene	7.16	0.006867719	3.013722757	1.706408203	up	C07111
acetyl-l-carnitine	3.354	0.013115768	0.312472405	1.671435622	down	C02571
l-tyrosine	3.991	0.00149854	0.386165572	1.654585078	down	C00082
porphobilinogen	3.352	0.034868003	0.20216218	1.654454946	down	C00931
l-threonic acid	0.943	0.002657091	0.347409401	1.642131651	down	C01620
4-acetamidobutanoic acid	1.668	0.000570632	0.436407661	1.61978909	down	C02946
4-acetamido-2-amino-6-nitrotoluene	3.263	0.00111977	0.227842697	1.608876089	down	C16420
glycocholic acid	4.221	0.014936314	0.103336992	1.60597848	down	C01921
dihydrozeatin-o-glucoside	1.61	0.01792962	0.271406689	1.604519415	down	C16448
11-cis-retinol	7.163	0.006451692	2.915628763	1.572908705	up	C00899
nicotinamide	3.424	0.007614706	5.146332097	1.547648262	up	C00153
isobutyric acid	2.941	0.005541458	0.388839906	1.535003163	down	C02632
lysopc(20:5(5z,8z,11z,14z,17z))	5.263	0.027957982	2.832175584	1.526168548	up	C04230
indole	7.164	0.011630975	2.35264951	1.519588697	up	C00463
dl-serine	1.356	0.023690522	2.540369095	1.509865627	up	C00740
6-hydroxynicotinic acid	2.273	0.002869619	0.328312222	1.493237216	down	C01020
2-oxindole	3.948	0.01723703	2.154561732	1.487481751	up	C12312
carglumic acid	0.876	0.004993102	0.324537023	1.459809606	down	C05829
felbamate	3.397	0.031618975	0.326616023	1.459068561	down	C07501
(-)-beta-pinene	7.161	0.003542711	2.558699424	1.439075543	up	C06307
caprolactam	1.544	0.036225751	0.372001375	1.427223338	down	C06593
coumarin	1.039	0.004518272	0.34506052	1.426590716	down	C05851
1-palmitoylglycerophosphocholine	7.36	0.016329714	2.084945478	1.421688962	up	C04230
(r)-prunasin	3.661	0.028985834	0.444901788	1.38021666	down	C00844
daidzein	4.131	0.028844658	2.139920006	1.36044792	up	C10208
8(s)-hydroxy-(5z,9e,11z,14z)-eicosatetraenoic acid	5.901	0.035044225	2.768271739	1.354150674	up	C14776
1-nitro-7-hydroxy-8-glutathionyl-7,8-dihydronaphthalene	3.669	0.019572094	0.010086674	1.34385978	down	C14803
p-cymene	5.866	0.005126214	2.652075636	1.333584526	up	C06575
solanidine	8.572	0.003967848	2.138868299	1.328504658	up	C06543

paraxanthine	1.67	0.015770188	0.387223304	1.305001813	down	C13747
mevalonic acid	2.886	0.003700736	0.448910756	1.280227497	down	C00418
1-pyrroline-4-hydroxy-2-carboxylate	3.656	0.034032524	0.284070453	1.279618758	down	C04282
taurine	0.885	0.004452054	7.259391859	1.278225712	up	C00245
picolinic acid	1.507	0.01480599	0.401570518	1.277151832	down	C10164
3-methyldioxyindole	3.993	0.000904988	0.425342413	1.266505067	down	C05834
guanine	3.227	0.038725055	0.42917386	1.260403889	down	C00242
n-acetylneuraminic acid	0.947	0.013747327	2.334360933	1.254603246	up	C00270
4-hydroxybenzaldehyde	1.688	0.010291693	0.40137665	1.251762595	down	C00633
acetophenone	4.879	0.026044417	2.942563674	1.249821054	up	C07113
capsidiol	4.074	0.026823681	2.539396658	1.247220955	up	C09627
5-hydroxyindoleacetylglycine	3.958	0.001189712	2.167830969	1.218836526	up	C05832
norepinephrine	1.597	0.016210031	0.35430396	1.199885714	down	C00547
genistein	4.343	0.002679066	3.845061752	1.189749758	up	C06563
benzamide	4.302	0.043074678	0.345033341	1.182040461	down	C09815
2-hydroxycinnamic acid	1.688	0.010722368	0.39995345	1.160592681	down	C01772
capecitabine	3.427	0.000219385	0.445039195	1.159982423	down	C12650
n-acetylhistamine	1.096	0.01653232	3.128434144	1.159731709	up	C05135
inosine	3.271	0.011946793	0.31947158	1.15603929	down	C00294
phenol	1.675	0.009739943	0.399609517	1.142936247	down	C00146
methylimidazoleacetic acid	0.938	0.000981813	2.01320934	1.138124817	up	C05828
1-methylxanthine	3.237	0.002974461	0.268825032	1.111256933	down	C16358
4-methyl-5-thiazoleethanol	3.174	0.039753886	0.443751838	1.080488951	down	C04294
1-nitrosonaphthalene	3.84	0.011130714	2.626955731	1.078626627	up	C14788
xanthine	3.276	0.008506335	0.401240835	1.067549564	down	C00385
2-keto-4-methylthiobutyric acid	3.399	0.00429091	0.370947371	1.060908671	down	C01180
creatine	0.841	0.006996609	2.18416189	1.047402985	up	C00300
sulfate	1.058	0.009720896	2.196831754	1.028896734	up	C00059
butanal	1.82	0.020098888	2.085890151	1.011801619	up	C01412
**P70 FS**						
phloionolic acid	4.843	0.009219224	8.338794627	3.75184514	up	C19621
ascorbic acid	3.294	0.000925818	223.5193785	3.669694051	up	C00072
11-cis-retinol	9.652	0.00348762	7.915887305	3.298159787	up	C00899
l-histidine	3.821	0.000507195	0.186511666	3.213315029	down	C00135
glycochenodeoxycholic acid	4.925	0.001548887	4.221176036	2.831071936	up	C05466
lotaustralin	3.709	0.013026729	0.438095103	2.76282098	down	C08334
10-hydroxycarbazepine	4.516	0.038931982	2.246186075	2.712596544	up	C07493
stearic acid	6.545	3.33E-05	5.982708442	2.701933513	up	C01530
cinnamyl alcohol	5.615	0.002382847	2.544820942	2.619914701	up	C02394
1-palmitoylglycerophosphocholine	4.572	0.003749397	8.048275727	2.544082337	up	C04230
5-phosphonooxy-l-lysine	0.851	0.00138809	0.469351539	2.526656485	down	C03366
homogentisic acid	3.532	0.016578156	0.38554631	2.517979649	down	C00544
acetyl-l-carnitine	0.9	0.048936619	0.371320604	2.501695896	down	C02571
4-acetamidobutanoic acid	0.871	0.02022881	0.481705111	2.406410338	down	C02946

4-(methylnitrosamino)-1-(3-pyridyl-n-oxide)-1-butanol	3.208	0.011915853	0.26625409	2.346855252	down	C19603
porphobilinogen	5.092	0.003487682	11.21925757	2.262258405	up	C00931
6-ketoprostaglandin f1 伪	4.692	0.003275903	0.481212718	2.255877096	down	C05961
phenol	3.423	0.044147008	2.187211605	2.24689239	up	C00146
styrene	5.614	0.007468907	2.859987297	2.221769288	up	C07083
d-proline	3.379	0.013547516	0.389082166	2.207445333	down	C00763
dl-serine	1.356	0.001488988	3.707988694	2.132975045	up	C00740
n-formyl-l-aspartate	0.954	0.000682896	0.471255768	2.130598153	down	C01044
costunolide	4.014	0.017066369	0.413559284	2.051195864	down	C09382
tetrandrine	4.972	0.025098361	4.964264684	2.049027318	up	C09654
linoleic acid	6.54	0.039101228	5.055056427	2.033228854	up	C01595
histamine	0.804	0.04842457	2.419784041	1.993826301	up	C00388
(s)-2-acetolactate	0.942	0.021177843	0.389244828	1.983795114	down	C06010
pantothenol	1.512	0.04419887	0.116383454	1.93965008	down	C05944
indole	5.62	0.003265269	3.007764406	1.932885778	up	C00463
adenosine	3.279	0.017429451	8.042391193	1.920976932	up	C00212
5beta-cyprinolsulfate	4.904	0.000682335	5.354133339	1.920556252	up	C05468
3-dehydroteasterone	4.577	0.009141296	3.256413724	1.904819674	up	C15792
n-succinyl-2-l-amino-6-oxoheptanedioate	3.351	0.004252545	0.399741467	1.870900183	down	C04462
chenodeoxyglycocholate	4.969	0.005802732	5.182466671	1.858328325	up	C05466
ethylbenzene	4.468	0.000798805	2.149200244	1.858254085	up	C07111
cafeine	0.894	0.027098166	0.343045493	1.834596246	down	C07481
20alpha-hydroxy-4-pregnen-3-one	4.568	0.002126352	2.6251457	1.81767132	up	C04042
ethoxyquin	8.107	0.034666068	2.48906766	1.787022374	up	C07475
caprolactam	3.274	1.66E-05	2.596524782	1.761418373	up	C06593
pregnenolone	4.446	0.014362926	2.420637155	1.758806541	up	C01953
n-acetylhistamine	1.096	0.049324591	2.142485976	1.721331579	up	C05135
d-arabinonate	0.911	0.004461015	0.362932665	1.713460487	down	C00878
8z,11z,14z-eicosatrienoic acid	9.217	0.022005549	7.27790085	1.697414933	up	C03242
cuminaldehyde	5.629	0.003130143	2.136341527	1.688053512	up	C06577
l-kynurenine	1.505	0.006732238	2.286371689	1.63642295	up	C00328
l-tyrosine	1.686	0.040459977	2.189206621	1.633265552	up	C00082
tryptamine	3.528	0.030845462	3.564751328	1.627817142	up	C00398
l-isoleucine	3.38	0.000513943	2.138866616	1.614009222	up	C00407
(-)-trans-carveol	4.092	0.041861525	0.45295412	1.611245235	down	C00964
p-cymene	5.615	0.008837824	2.376304227	1.604401389	up	C06575
palmitic acid	5.875	0.03160947	0.287371449	1.590952281	down	C00249
11-cis-retinal	6.859	0.021505216	0.447828362	1.553719939	down	C02110
l-aspartic acid	0.876	0.020973361	3.664885297	1.532451875	up	C00049
4-imidazolone-5-propanoate	0.933	0.049404103	2.214920531	1.523365852	up	C03680
capsidiol	4.029	0.015560632	0.456243993	1.494452492	down	C09627
deoxycholic acid	5.09	0.000822503	3.229361492	1.49336431	up	C04483
arachidonic acid	6.106	0.028798624	2.036967146	1.489066679	up	C00219
adenine	1.085	0.036585007	2.696289722	1.463023835	up	C00147
phenylglyoxylic acid	3.561	0.025683208	0.352979575	1.459926263	down	C02137
melatonin	5.051	0.018832837	0.474766657	1.453056867	down	C01598

benzamide	1.617	0.036803471	2.111626452	1.4432232	up	C09815
lysopc(p-18:0)	7.359	0.031726342	2.125970845	1.440222695	up	C04230
norsanguinarine	3.88	0.047083626	0.302562857	1.422676614	down	C05191
1-pyrroline-4-hydroxy-2-carboxylate	4.064	0.000285346	3.212345233	1.414429279	up	C04282
taurine	0.885	0.004565652	5.527253518	1.394040877	up	C00245
sn-glycero-3-phosphoethanolamine	0.884	0.020371829	0.470304913	1.378979309	down	C01233
d-sphingosine	4.964	0.029333768	2.034220751	1.354065701	up	C00319
gamma-tocotrienol	5.172	0.029114845	2.123254907	1.347349746	up	C14155
d-serine	0.879	0.004152209	2.577477422	1.334413562	up	C00740
glycocholic acid	4.428	0.020095241	6.903357615	1.290228406	up	C01921
skatole	3.782	0.025839613	3.912163296	1.278171981	up	C08313
kaempferol	4.153	0.048373802	2.170203342	1.255984842	up	C05903
glutaric acid	2.854	0.013525687	0.256531466	1.234846967	down	C00489
4-methylcatechol	3.513	0.032327095	0.495563138	1.222137186	down	C06730
thymidine	3.879	0.017239626	0.451627528	1.213473833	down	C00214
diethanolamine	5.343	0.046718876	0.264470161	1.207249551	down	C06772
cholic acid	4.246	0.008141833	2.201871056	1.167304637	up	C00695
2-keto-4-methylthiobutyric acid	3.399	0.049483263	0.352077937	1.074840262	down	C01180
l-muricholic acid	4.456	0.007740699	2.305213612	1.047367529	up	C17726
atrazine-desethyl	3.964	0.022491516	2.497705426	1.003439244	up	C06559
**P70 IC**						
cuminaldehyde	7.202	0.000747113	21.95850414	3.435233993	up	C06577
cytosine	3.188	0.015769574	0.069192586	2.669258898	down	C00380
biotin	3.665	0.011755897	2.056776616	2.64039848	up	C00120
ethylbenzene	8.36	0.017665874	5.141886793	2.629552762	up	C07111
d-glucono-1,5-lactone	0.97	0.015234094	2.647813984	2.531450756	up	C00198
diethanolamine	5.343	0.00690307	0.045145752	2.52722737	down	C06772
3-dehydroxycarnitine	0.998	0.013597525	3.688495344	2.474816837	up	C01181
8(s)-hydroxy-(5z,9e,11z,14z)-eicosatetraenoic acid	5.901	8.99E-05	8.507957851	2.425400402	up	C14776
trigonelline	0.964	0.036954816	3.662404732	2.386429636	up	C01004
i-urobilinogen	4.567	0.000320951	0.10100142	2.385223756	down	C05790
prostaglandin j2	4.746	0.007833343	8.291729674	2.379606966	up	C05957
4-fumarylacetoacetate	0.981	0.004030684	2.008703547	2.355939873	up	C01061
l-cystine	0.858	0.008761822	9.237955552	2.318443015	up	C00491
epinephrine	3.784	0.002506182	2.318048737	2.287288101	up	C00788
7-hydroxy-6-methyl-8-ribityllumazine	0.937	0.001433283	3.725521489	2.27985727	up	C05995
4-(beta-acetylaminoethyl)imidazole	3.34	0.000396821	2.807368216	2.23957348	up	C05135
d-sphingosine	4.672	0.025002436	0.288575407	2.116347641	down	C00319
(r)-lactate	0.928	0.036269826	2.479438768	2.096365255	up	C00256
n-acetyl-l-aspartic acid	1.488	0.013618463	4.315261396	2.081955862	up	C01042
indole-3-acetic acid	4.135	0.047981531	3.90953619	2.071500362	up	C00954
gentisic acid	3.403	0.000494761	8.278110873	2.038411511	up	C00628
8-amino-7-oxononanoate	4.013	0.001244589	2.591957995	2.035984521	up	C01092
caprolactam	3.638	0.004599902	3.790007073	2.031689642	up	C06593
methylimidazoleacetalde	0.996	0.038364814	2.09357138	2.020283856	up	C05827

hyde						
4-hydroxy-2-oxoglutarate	0.947	0.003859845	2.481363669	2.014167697	up	C01127
lysopc(p-18:0)	6.527	0.018760959	0.378745369	2.01268269	down	C04230
mesaconic acid	0.951	0.004375295	2.384583076	2.008810599	up	C01732
folic acid	3.424	0.002002554	7.042759463	2.008678511	up	C00504
acrylic acid	0.93	0.026142815	3.019623799	1.991518378	up	C00511
m-lactose	0.94	0.013677631	2.897339454	1.94456407	up	C00243
n-acetylputrescine	0.974	0.021401801	3.204871954	1.931572042	up	C02714
d-(+)-maltose	0.939	0.037299673	2.653033783	1.925336411	up	C00208
phloionolic acid	4.356	0.029330073	0.271873398	1.919812324	down	C19621
l-isoleucine	3.683	0.033208412	3.661929912	1.918933102	up	C00407
cortolone	4.325	0.024083021	2.851528947	1.914337043	up	C05481
5beta-cyprinolsulfate	5.274	0.005100879	0.056155711	1.89749994	down	C05468
gibberellin a53	4.621	0.002613859	3.049440921	1.865763489	up	C06094
naphthalene	4.817	0.038324142	0.275544689	1.861737441	down	C00829
phenol	1.035	0.012279829	0.42702987	1.84043615	down	C00146
6-hydroxypseudooxynicotine	3.456	0.01538148	0.351591855	1.808933581	down	C01297
l-histidine	3.821	0.010561824	0.39900753	1.807835018	down	C00135
hippuric acid	3.765	0.049014692	0.13180995	1.777239237	down	C01586
acetyl-l-carnitine	3.437	0.008442397	2.868939354	1.766688812	up	C02571
dimethylbenzimidazole	3.371	0.010604932	3.094931799	1.705430396	up	C03114
daidzein	4.131	0.000598771	3.505788996	1.705048918	up	C10208
agmatine	0.81	0.024066097	0.168992048	1.698414661	down	C00179
prostaglandin e2	4.188	0.002407624	2.193753723	1.696413459	up	C00584
mivacurium	4.338	0.004852981	0.151117334	1.693774204	down	C07550
testosterone	5.619	0.026869079	2.244619044	1.685401204	up	C00535
pregnenolone	6.006	0.033026353	3.294164229	1.681632374	up	C01953
salicylic acid	3.617	0.016855017	2.682803794	1.657963788	up	C00805
palmitoylcarnitine	5.222	0.046557047	0.395134517	1.657796698	down	C02990
naringenin	4.291	0.001184771	0.09581972	1.653996973	down	C00509
11-cis-retinol	7.439	0.009940921	0.329270275	1.652887547	down	C00899
d-mannose 6-phosphate	0.888	0.005261262	2.591557598	1.64915558	up	C00275
4-anisic acid	3.5	0.00069364	2.288555044	1.648003633	up	C02519
berberine	3.861	0.038320899	0.440995851	1.643817381	down	C00757
pantothenic acid	3.375	0.00163613	2.079821169	1.628305647	up	C00864
capsaicin	3.833	0.008785463	2.141122938	1.624415558	up	C06866
capsidiol	8.223	0.00690354	4.236082897	1.611109404	up	C09627
pilocarpine	4.298	0.022201199	0.116824783	1.602176957	down	C07474
cyclohexanecarboxylic acid	3.964	0.048456956	2.493405252	1.590179037	up	C09822
jasmonic acid	5.483	0.000978139	3.106926567	1.56192032	up	C08491
5-oxoete	5.483	0.024620019	3.361211029	1.561825672	up	C14732
apc	4.057	0.001267272	0.385366706	1.5612844	down	C16542
4-acetamidobutanoic acid	3.522	0.004558452	2.398549194	1.560106668	up	C02946
arachidonic acid	6.106	0.007184436	0.417181941	1.541614113	down	C00219
taurine	4.343	0.002233217	0.097580956	1.538536479	down	C00245
indole	7.164	0.035502205	2.478070876	1.517043696	up	C00463
tryptamine	3.528	0.01854519	4.825122775	1.510193511	up	C00398

d-glucaric acid	0.915	0.021986876	6.363735543	1.50641814	up	C00818
phenylacetaldehyde	1.021	0.045425876	0.481821376	1.499851204	down	C00601
phenylglyoxylic acid	3.438	0.013429307	3.082832949	1.486214496	up	C02137
1-alpha-d-galactosyl-myo-inositol	1.495	0.009099363	2.069095864	1.474287657	up	C01235
d-arabitol	0.922	0.012866409	2.151616961	1.463528094	up	C01904
xanthohumol	5.45	0.029427084	2.595914093	1.462435158	up	C16417
i-urobilin	4.536	0.006693825	0.248289347	1.454297209	down	C05794
ascorbic acid	4.521	0.018857595	3.346384978	1.448296689	up	C00072
6-gingerol	5.415	0.030019556	0.461463726	1.435763032	down	C10462
20alpha-hydroxy-4-pregnen-3-one	6.425	0.027616929	2.713532743	1.420834153	up	C04042
homovanillic acid	3.591	0.02031994	0.345056166	1.408333587	down	C05582
norepinephrine	1.597	0.0308221	0.367774115	1.406732351	down	C00547
kynurenic acid	4.176	0.019834262	0.300513797	1.379517807	down	C01717
3-methoxytyramine	4.023	0.008690418	0.293849802	1.368907371	down	C05587
oxalosuccinate	0.984	0.038862633	2.772701054	1.339773932	up	C05379
benzamide	4.302	0.010924496	3.245862915	1.336130649	up	C09815
d-arabinonate	0.911	0.003999756	2.239347195	1.328943761	up	C00878
alpha-tocotrienol	4.295	0.038289715	0.471933669	1.326223093	down	C14153
styrene	4.816	0.040547849	0.354762612	1.317192309	down	C07083
2-arachidonoylglycerol	8.99	0.016623425	3.264300362	1.314445086	up	C13856
cortol	5.841	0.007848987	2.456601735	1.306460313	up	C05482
docosapentaenoic acid	5.008	0.013525967	0.464628078	1.302897311	down	C16513
2-hydroxycinnamic acid	1.688	0.048496869	0.384159467	1.290676476	down	C01772
orotic acid	1.393	0.003129049	0.366034786	1.272509167	down	C00295
corticosterone	6.749	0.026426988	4.821395024	1.261753874	up	C02140
taurochenodeoxycholic acid	4.797	0.039977292	0.066569742	1.259267661	down	C05465
m-trehalose	0.939	0.020704831	2.692147321	1.253222522	up	C01083
4-imidazolone-5-propanoate	0.938	0.013566111	2.205615168	1.238396863	up	C03680
6-pyruvoyltetrahydropterin	3.362	0.029493078	2.012593633	1.234975694	up	C03684
3-dehydroteasterone	4.577	0.001746719	0.457996169	1.203663208	down	C15792
[6]-gingerol	4.957	0.043931259	2.111764234	1.203129499	up	C10462
paraxanthine	1.67	0.029057357	0.471738023	1.199701084	down	C13747
5-hydroxyindole-3-acetic acid	3.796	0.037398586	0.337511568	1.194943737	down	C05635
1-methoxypyrene	3.449	0.040572127	2.551032029	1.186864812	up	C18259
coumarin	3.644	0.044743555	0.373858989	1.156888434	down	C05851
2-keto-4-methylthiobutyric acid	3.399	0.001189841	2.922913054	1.136183209	up	C01180
geosmin	4.876	0.003044977	0.446471364	1.116935377	down	C16286
acetophenone	1.704	0.041397019	0.42025066	1.109935868	down	C07113
3-methoxy-4-hydroxyphenylethyleneglycol	4.012	0.033799456	0.392518706	1.109695553	down	C05594
genistein	3.988	0.041678993	3.545963677	1.107864807	up	C06563
d-(+)-xylose	0.94	0.00724313	2.42011404	1.088691587	up	C00181
cholesterol sulfate	4.452	0.004006662	0.2693033	1.087261506	down	C18043
glycocholic acid	4.428	0.002774131	0.240045732	1.012182893	down	C01921
2-coumarate	3.566	0.014752488	0.35622507	1.002366693	down	C01772

RT, retention time. VIP, PLS-DA variable projection importance of the model. Fold Changes, different multiples of

univariate analysis. Regulated, the tendency of change compared with the control group. KEGG, codes annotated to the KEGG database. FS, feces. IC, ileal contents.
